# Disaggregated analysis of birth averted due to family planning use in India: An evidence from NFHS-4 (2015–16)

**DOI:** 10.1371/journal.pone.0239376

**Published:** 2020-09-23

**Authors:** Ujjaval Srivastava, Kaushalendra Kumar Singh, Pawan Kumar Yadav

**Affiliations:** 1 Department of Statistics, Banaras Hindu University, Varanasi, India; 2 National Statistical System Training Academy, Ministry of Statistics & Programme Implementation, Government of India, India; 3 International Institute of Population Sciences, Mumbai, India; Keele University, UNITED KINGDOM

## Abstract

**Background and objective:**

India contributes a major share of global unintended births. It is established that contraception plays a significant role in preventing unintended pregnancies, maternal mortality and induced abortion. In this study, to analyze the effectiveness of our family welfare program, we tried to give district-level estimates of number of births averted due to contraception.

**Data and methods:**

Data for this study came from the cross-sectional, population-based data from the fourth round of National Family Health Survey (NFHS-4) conducted in 2015–16. Here, we discussed two methods based on robust regression for computing number of births averted at district level. Further, we analyzed the percentage increase in births (PIB) that would be experienced by each district in the absence of contraception.

**Results:**

Findings of this study clearly showed that there was a huge variation in the estimates of number of births averted among different districts as well as states of India. Out of 640 districts, 315 districts achieved below-replacement fertility and 365 districts have contraceptive prevalence rate (CPR) more than 50 percent. Method 1 found around 22 percent districts showed less than 15 percent reduction in births while Method 2 suggested nearly 14 percent districts predominantly located in Uttar Pradesh, Bihar, Jharkhand, Arunachal Pradesh, Meghalaya and Manipur exhibited less than 30 percent reduction of births due to use of all forms of contraception. At all India level, an average estimate obtained by two methods, nearly 63 million births would have been averted by the use of contraception or 40 percent more than the number of births occurred during that period.

**Conclusion:**

The study successfully identified the districts that were not performing well at the front of utilization of various family planning methods for birth control. To achieve objectives of National Population Policy (2000), poor-performing districts must be monitored like the government keeps monitoring of *Aspirational districts*.

## Introduction

### Background

India is the 2nd most populous country in the world after China. The 2017 Revision of World Population Prospects said that “*In roughly seven years*, *or around 2024*, *the population of India is expected to surpass that of China*” [1]. The problem of the fast-growing population is viewed as creating obstructions for development. Birth control appears to be the most suitable method for the control of a growing community. In this context, social activist Raghunath Dhondo Karve is the first person who thought of opening a birth control clinic in Mumbai in the 1920s [2]. Afterwards, Independent India recognized the importance of family planning and became the first country in the world to initiate the National Family Planning Program in 1952. The principal objective behind this family planning program was to control the prompt growth of the population. Many approaches have been used by the government for promoting and use of various family planning methods, especially for those families where the family size is large. To evaluate the usefulness of these family planning programs for the control of fertility and to take steps for making these programs more efficient, each country wants to analyze that how many births are avoided attributable to the use of contraception in their country.

National Family Health Survey (NFHS) data shows that in 1992–93, the contraceptive prevalence rate among married/in-union women of reproductive age was 40.7 percent and it has risen to 53.5 percent in 2014–15 [3–7]. Still, the proportion of women having unintended and mistimed births is 5 and 4 percent respectively, which is a significant figure in absolute terms [7]. A large number of unwanted pregnancies results in induced abortions. The unsafe practice of induced abortion can be risky for the life of the mother. It is evident from the established literature that use of conceptive can reduce the risk of unintended pregnancies, induced abortions and maternal mortality [8]. Data shows a significant reduction in the national total fertility rate from 3.4 in 1992–93 to 2.2 in 2014–15 [4–7]. With all efforts taken by the government and other NGOs in the country, India is close to achieving replacement level fertility. However, in a culturally and demographically diversified country like India, the visible national progress may camouflage the disparities at state and local levels. Therefore, it is necessary to know how effective our family planning programs are at the local level. However, to our knowledge, no research study found till now, which tried to give the same estimates for below states/regional or district level. Therefore, this study aimed to provide estimates of the number of births averted due to contraception for 640 districts of India using NFHS-4 data.

### Scientific literature

From the late 1960s, many researchers tried to estimate the births averted due to different contraceptive methods for the evaluation of family planning programs [9–20]. Initially, some selected rules of thumb were widely used in which based on previously available pieces of evidence, demographers assign some assumed proportion of birth averted for particular contraceptive to the specific location. Keeny et al. [21] used Potter’s result on Taiwanese women as a standard rule of thumb which states that each IUD prevents 0.64 births in Taiwan. Mauldin [9] suggested that one can think of at least five approaches (based on marital age-specific fertility rates) for getting an answer to “how many births have been avoided as a result of any family planning program”. He emphasized on to see the difference in the rate of decline in fertility between case group (who exposed to family planning) and control group (non-users of contraceptives but matched for other socio-economic characteristics of case group). Wolfers [10] used concepts of life table to calculate birth averted. Potter’s method [16] used couple years of effective contraception which is one of the most elaborate method. It was based on delaying women’s stay in fecundable state due to the use of contraceptives, but it requires data on the expected length of the birth interval in the absence of contraception and duration of expected interruption of childbearing. Again, Potter [17] used the renewal theory model to estimate the expected number of births averted. Later Kelly [11] highlighted the disadvantages of methods based on age-specific fertility rates (ASFR). It was impracticable to estimate ASFR before and after the family planning program, directly from the population in less developed countries. Therefore, Kelly devised ‘parity approach’ to calculate ASFR from parity data which is readily available in both developing as well as developed countries. Venkatacharya [12] also derived a matrix (each for IUCD, salpingectomy and vasectomy) of annual probabilities of births specific to age at the start of contraceptive method and duration since adoption. These matrices were used to obtain the number of births averted due to IUCDs and sterilizations in India during 1956–69.

Again, Venkatacharya & Das [13] used a Monte-Carlo model to estimate the number of birth averted in India due to sterilizations during 1956–67 and IUCDs during 1965–67. Further, Bhattacharya et al. [18] derived a probability model for the number of births to a couple in first *T* years of marriage where *T* is large, and the couple is using various family planning methods. Then this model is used to illustrate the changes in the childbearing pattern due to contraception. A large number of further development can be found in series of manuals of United Nations (e.g. Trend analysis, experimental designs, Couple-years of protection, Experimental designs, Regression analysis, Simulation models, prevalence method etc.) to measure the impact of contraception on fertility. The data requirement, assumptions, limitations and pros & cons of each approach are mentioned in *Manual IX* [14, 15]. After a lag of 2 decades, Liu et al. [19] proposed three methods to compute global and regional estimates of births averted due to contraception for 156 countries during 1986–2004: (1) Robust regression of General Fertility Rate (GFR) and Contraceptive Prevalence Rate (CPR) among currently married women (CMW) with a linear and quadratic term of CPR (CMW) as independent variables; (2) Instead of GFR, Total Fertility Rate (TFR) is chosen as the dependent variable; (3) the Third approach is based on Bongaarts’ proximate determinants of fertility [22]. Further, Singh et al. [20] attempted to produce estimates of births averted attributable to contraception for 19 major states of India. This method is based on the observed relationship between TFR and New Predictor Variable (NPV) which is a combination of CPR and proportion of currently married women having open birth interval more than five years and are not using any contraceptives during last five years. Later Rai et al. [23] also tried to give estimates of birth averted due to different family planning methods for 29 states of India using 3rd round of National Family Health Survey (NFHS) data.

## Data and methods

### Ethics statement

Present study was based on a large dataset that is publicly available on DHS website (https://dhsprogram.com/data/) conducted by the MoHFW and International Institute for Population Sciences (IIPS) in India with ethical standards being complied with including informed consent obtained from participants.

### Data and sample design

Data for this study was taken from the most recent round of NFHS -4 (Fourth round) conducted in 2015–16. The previous three rounds of NFHS were conducted in 1992–93, 1998–99 and 2004–05. NFHS is India’s largest household survey for health planning and policy formulation in the country. It is conducted by the Ministry of Health & Family Welfare (MoHFW), Government of India. NFHS-4 used a stratified two-stage sampling design to get a representative sample from the country. In total, 699,686 women of age group 15–49 years and 122,122 men of age group 15–54 years were interviewed through separate questionnaires using Computer Assisted Personal Interviewing method. It provides extensive information on key indicators of the utilization of maternal and child health services, nutrition, fertility, reproductive health, quality of family planning services, domestic violence, infant and child mortality etc. Besides these indicators, it also collects biomarkers information on haemoglobin, blood pressure, random blood glucose, height, weight and HIV. NFHS-4 is different from other previous rounds of NFHS-1, 2 & 3 since its overall sample size is decided in such a way that it can provide indicators at district, state/union territory and national levels. Additionally, NFHS-4 provides separate estimates for rural and urban areas of 157 districts where around 30–70 percent population residing in urban India as per 2011 census. For more details of survey design and questionnaires, one can refer to NFHS- 4 report [7]. In this article, for estimating district wise birth averted that is attributed to the use of any method of contraception (Modern and traditional methods), we applied a robust regression approach. It is pertinent to mention that the relationship of TFR and CPR studied through robust regression in this study, is under assumption that other proximate determinants will remain the same. Further, we assume that the current level of contraceptive prevalence prevailed within the reference period under study. Steps involved in calculations are given below:

#### Step I - Calculation of observed TFR and CPR for each district

We estimated TFR and its 95% confidence interval (CI) for each district of India. The reference period for the calculation of TFR was three years before survey, i.e. only births occurred within three years before the survey date were used to compute district-level TFR. Here, we calculated TFR directly from the available birth histories of the women. For this purpose, we used STATA *tfr2* module as described by Schoumaker [24].

NFHS collects information on the current use of family planning methods. CPR is one of the primary indicators which is defined as the percentage of currently married women of age group 15–49 years who are using any contraceptive methods (traditional or modern) or whose husband are using any contraception. We calculated CPR for each district directly from the NFHS-4 unit level data.

#### Step II—Calculation of potential TFR (TFRP) for each district

For the calculation of birth averted, our first task was to estimate TFRP which is defined as the TFR that would have been experienced by the currently married women of reproductive age group if they never used any method of contraception. So we regressed logarithm of observed TFR on observed CPR. The reason for choosing the logarithmic transformation of TFR was that the density of TFR was highly skewed and non-normal while the distribution of log of TFR and CPR were normal. Residual plot analysis also suggested that when we regress on CPR, the residuals were normally distributed. So, the two variable ordinary least-square (OLS) regression model was given by

$$\log{TFR}_{i}\;=\;\beta_{0}+\beta_{1}{CPR}_{i}+\varepsilon_{i\;}i\;=\;1,2,3\ldots ,N$$

**Fig 1:**
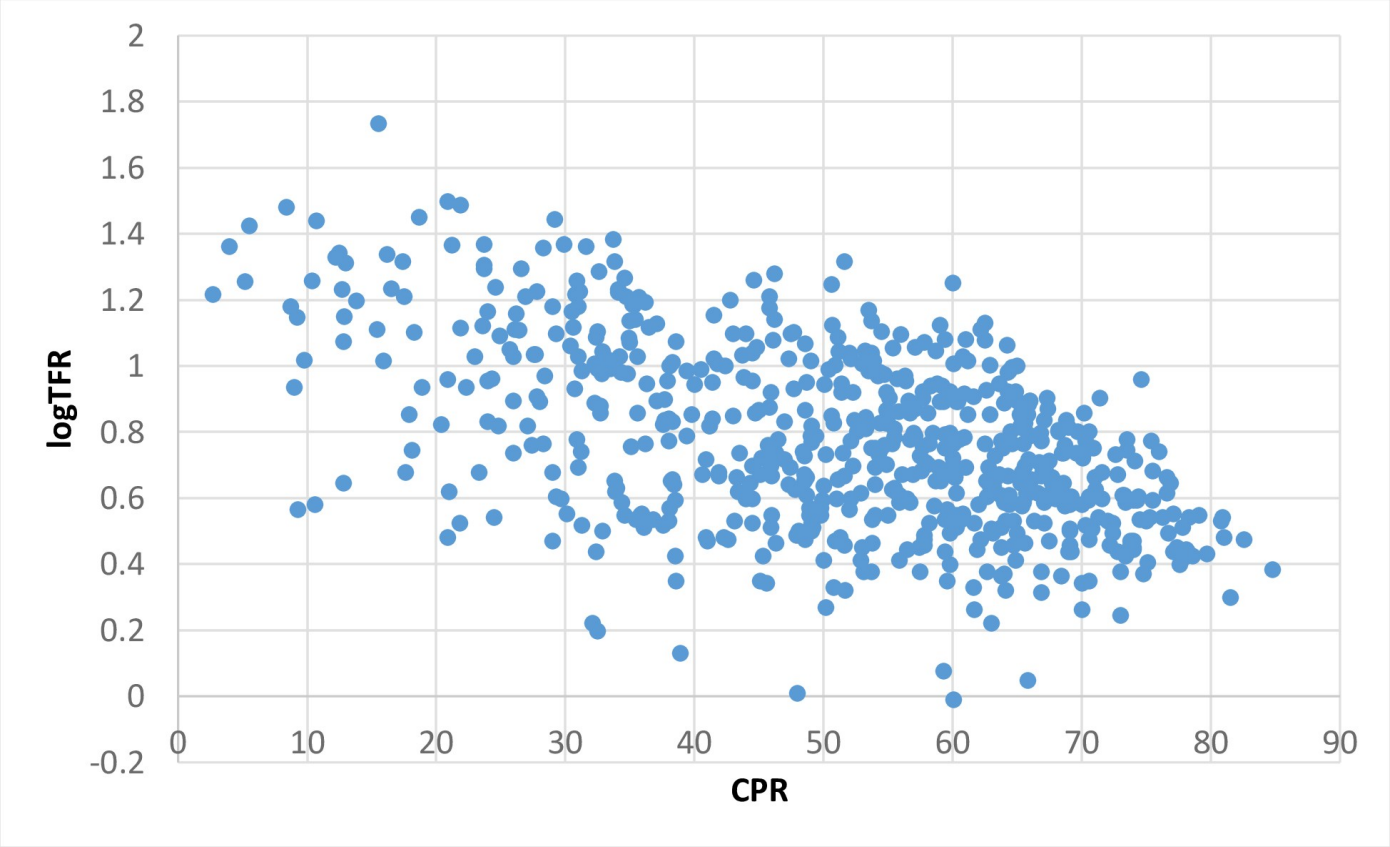
Scatterplot of logTFR versus CPR of districts of India.

Fig 1 shows the scatterplot of CPR versus the logarithm of TFR (actual or observed) for each district of India. Using the least square method, we can find estimates for$\;\beta_{0},\;\beta_{1}$ and ε. The scatterplot of and CPR showed an inverse relationship between these two variables, but there were some outliers present in our data. Outliers disturb the assumption of normally distributed residuals in ordinary least square regression and distort the values of regression coefficients by giving more weights than they deserve. Hence in the presence of outliers in the data, robust regression is a better alternative than OLS. In OLS, all the observations are given weights equal to 1 while in robust regression, the larger the value of residual associated with observation, the smaller the weight assigned to that observation in the regression.

The estimated robust regression model will be

$$\log{TFR}_{i}\;=\;\hat{\beta_{0}}+\hat{\beta_{1}}{CPR}_{i}+\hat{\varepsilon_{i}}\;i\;=\;1,2,3\ldots ,N$$

Where $\hat{\beta_{0}}$ denotes the average value of logTFR in the absence of any contraception and $\hat{\beta_{1}}$ shows the change in the mean value of logTFR corresponding to per unit change in the CPR and ε is disturbance term having some specific probability distribution. Here N is the total number of districts in India as per 2011 census which is equal to 640.

Using empirical data, $\hat{\varepsilon_{i}}$ can be easily calculated i.e. $\hat{\varepsilon_{i}}\;=\log{TFR}_{i}-(\hat{\beta_{0}}+\hat{\beta_{1}}{CPR}_{i})$. Finally, in the absence of CPR or we can say that if contraceptive use is absent in women/man between age group 15–49, then for different values of $\hat{\varepsilon_{i}}$, the possible value of log${TFR}_{i}$ would be equivalent to $\hat{\beta_{0}}+\hat{\varepsilon_{i}}$. We termed log${TFR}_{i}$ in absence of CPR as $\log{TFRP}_{i}\;$(loagaritm of Potential TFR). Therefore, it can be easily calculated. Thereafter, TFRP for each district can be obtained by taking exponential transformation of $\log{TFRP}_{i}$.

#### Step III—Calculation of Birth Averted due to contraception (BA) and Percentage Increase in Births (PIB)

Birth averted due to contraception (BA per 1000) was calculated using following formula:

$${BA}_{i}\;=\;\frac{\left({TFRP}_{i}-{TFR}_{i}\right)}{{TFR}_{i}}*Total\;birth\;\left(in\;last\;3\;years\right)*1000\;i\;=\;1,2,3\ldots ,N$$

For the comparison among different districts, India with varying sizes of population, and the number of births, we also generated a percentage increase in birth (PIB) in the absence of any contraception. So for calculation of PIB, we used the following formula:

$${PIB}_{i}\;=\;\frac{\left({TFRP}_{i}-{TFR}_{i}\right)}{{TFR}_{i}}*100\;i\;=\;1,2,3\ldots ,N$$

These two formulas were adopted from Liu et al. [19]. In present study, we obtained two estimates for the number of birth averted due to contraception for each district.

### Method 1

For first estimate (Method 1) we classified each district of India into four homogeneous group based on following definition as given in Table 1. In other words, where the relationship between TFR and CPR was weak, we formed Group 1 (both CPR and TFR were low) and Group 4 (both CPR and TFR were high). On the other hand, where the relationship was strong, we formed Group 2 (high TFR and low CPR) and Group 4 (low TFR and high CPR). In method 1, we fitted four separate robust regressions based on the number of observations in the respective four groups defined below to obtain TFRP for each district and then applied step III described above to obtain BA per 1000 and PIB.

**Table 1:** Classification of districts based on their TFR and CPR level under Method 1.

Group	Number of Districts
TFR below National level (2.2) & CPR below 50 (Group 1)	111
TFR above National level (2.2) & CPR below 50 (Group 2)	164
TFR below National level (2.2) & CPR above 50 (Group 3)	248
TFR above National level (2.2) & CPR above 50 (Group 4)	117
Grand Total (*N*)	**640**

### Method 2

For the second estimate, we fitted only one robust regression based on all 640 observations to obtain TFRP for each district and followed the step III to get BA per 1000 and PIB.

For obtaining aggregated estimates at state level, first, we estimated number of BA at district level. Then these BA were summed over all districts to get state-level estimate of BA, and this estimate was divided by the total number of births in that state. The above procedure gave us PIB estimate for that state. A similar procedure was followed to obtain BA and PIB estimates at country level.

## Results

Under method 1, the separate OLS regression equation obtained for each class are given below:

For Group 1:

$$log{TFR}_{i}\;=\;0.601-\;0.00025*\;{CPR}_{i}\;i\;=\;1,2,\ldots ,111.$$

For Group 2:

$$log{TFR}_{i}\;=\;1.305\;-0.0069*\;{CPR}_{i}\;i\;=\;1,2,\ldots ,164.$$

For Group 3:

$$log{TFR}_{i}\;=\;0.735-0.0026*\;{CPR}_{i}\;i\;=\;1,2,\ldots ,248.$$

For Group 4:

$$log{TFR}_{i}\;=\;1.314-0.0063*\;{CPR}_{i}\;i\;=\;1,2,\ldots ,117.$$

After that adjusting for outliers for each defined class, we computed 4 separate robust regressions. The corresponding 4 equation for calculation of $\log{TFRP}_{i}$ are given below:

For Group 1:

$$log{TFRP}_{i}\;=\;log{TFR}_{i}+0.00061*\;{CPR}_{i}\;i\;=\;1,2,\ldots ,111.$$

For Group 2:

$$log{TFRP}_{i}\;=\;log{TFR}_{i}+0.0068*\;{CPR}_{i}\;i\;=\;1,2,\ldots ,164.$$

For Group 3:

$$log{TFRP}_{i}\;=\;log{TFR}_{i}+0.0034*\;{CPR}_{i}\;i\;=\;1,2,\ldots ,248.$$

**Fig 2:**
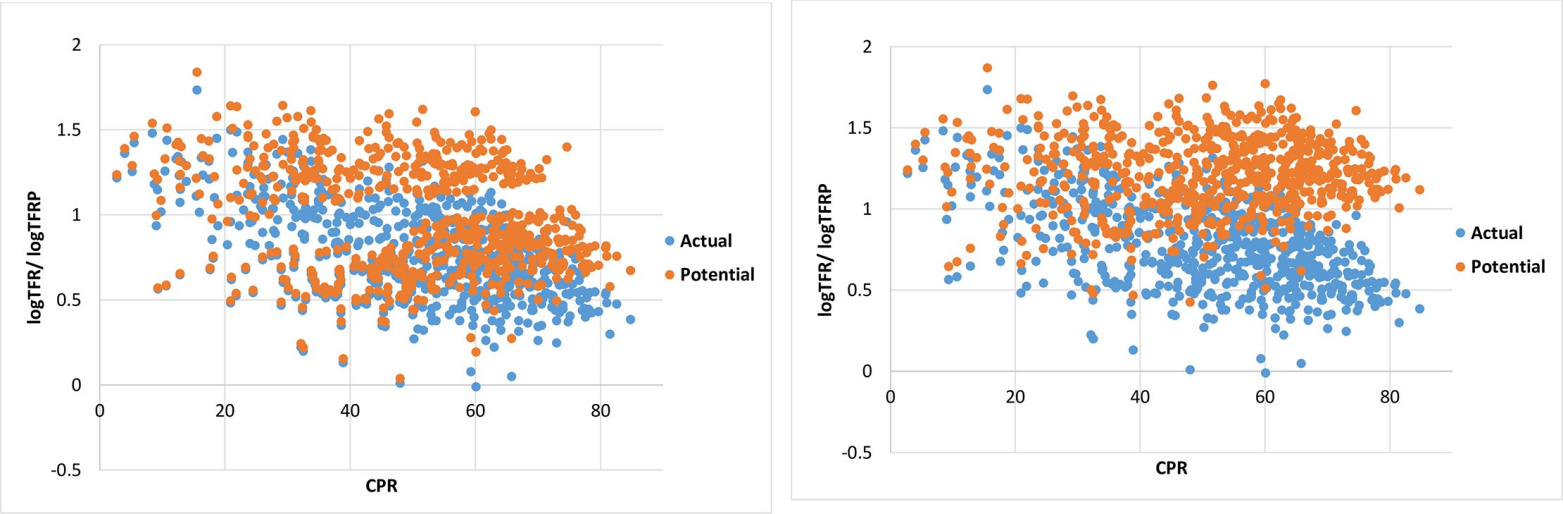
(a) Scatterplot of logTFR/ logTFRP versus CPR of districts of India under Method 1. (b) Scatterplot of logTFR/ logTFRP versus CPR of districts of India under Method 2.

For Group 4:

$$log{TFRP}_{i}\;=\;log{TFR}_{i}+0.0059*\;{CPR}_{i}\;i\;=\;1,2,\ldots ,117.$$

and then PIB and BA per 1000 were calculated as per above mentioned procedure. Fig 2(A) & 2(B) shows the scatterplot of logarithm of actual TFR / estimated TFRP for each district from the method 1 and method 2 respectively versus CPR of each district.

Under method 2, the OLS regression equation came out to be:

$$log{TFR}_{i}\;=\;1.199-0.0083*\;{CPR}_{i}\;i\;=\;1,2,\ldots ,640.$$

Thereafter adjusting for outliers, we computed robust regression based on 640 observation. The corresponding equation for the calculation of $\log{TFRP}_{i}$ iss given by:

$$log{TFRP}_{i}\;=\;log{TFR}_{i}+0.0087*\;{CPR}_{i}\;i\;=\;1,2,\ldots ,640.$$

Then rest procedure for the calculation of PIB and BA per 1000 was same as defined above in step III.

**Fig 3:**
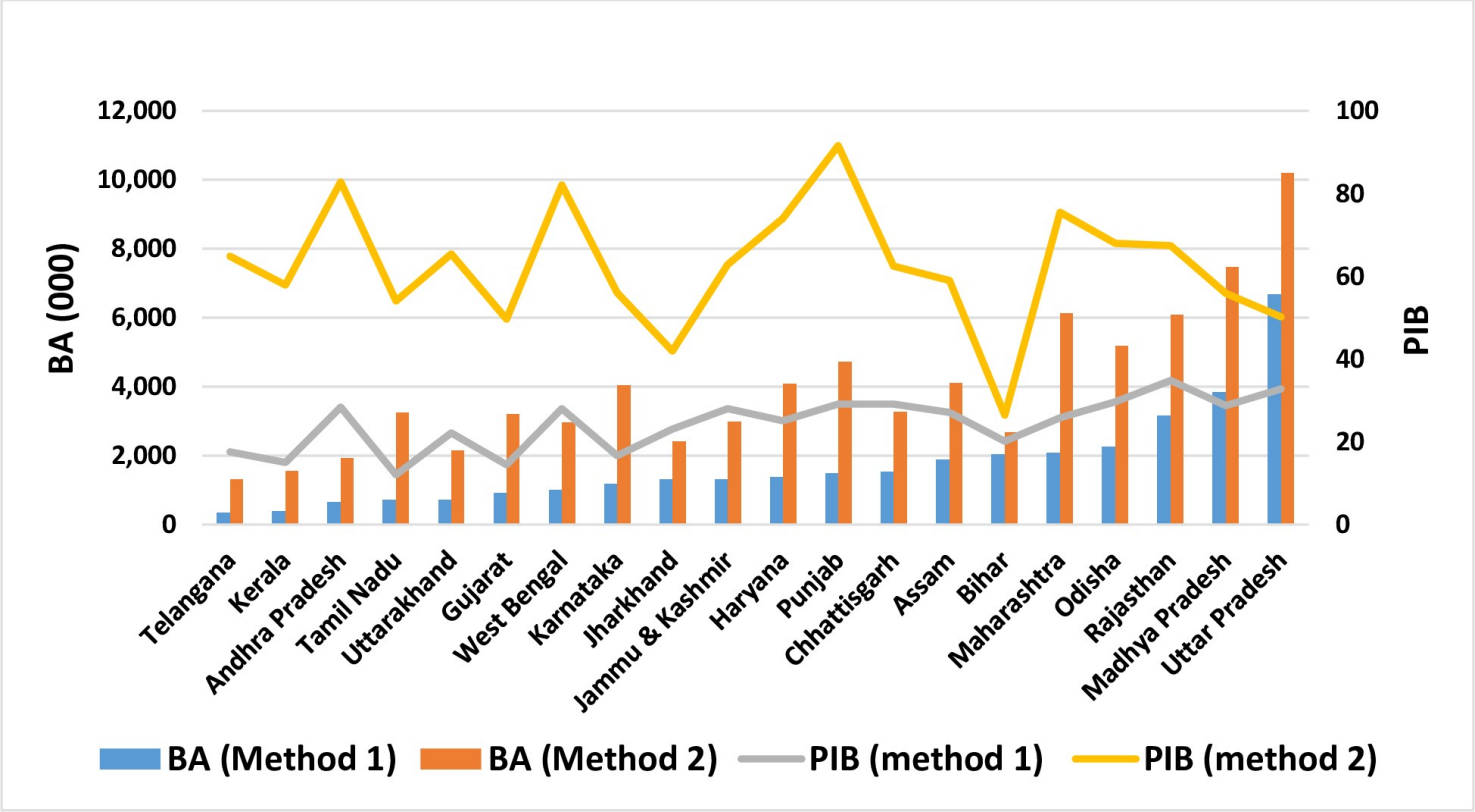
State wise estimates of BA, PIB for bigger states of India by the two methods for the three years preceding the survey.

Results of district-level estimates of birth averted and percentage increase in the births in the absence of any contraception for the above mentioned two approaches are shown in S1 Table. In contrast, the aggregated estimates for all the states, union territories and country are presented in Table 2. At the country level, the estimates of birth averted by contraception were 38 million with the first approach and 87 million with the second approach, which was more than two times of the first estimate. Analyzing the first approach among bigger states through Fig 3, we found that Uttar Pradesh had the highest number of birth averted (6.6 million) followed by Madhya Pradesh (3.8 million) and then Rajasthan (3.1 million) due to contraception. By the second approach, these numbers became higher than estimates obtained by the first approach. We found more than 10 million births were averted in Uttar Pradesh, followed by 7.4 million in Madhya Pradesh and 6.1 million in Maharashtra by using the second approach. From the bottom side, the newly constructed state, Telangana had the lowest number of births averted followed by Kerala and Andhra Pradesh by both methods. The gap between the two estimates of BA given by two different approaches was highest for Maharashtra (around 4 million) and the lowest for Bihar (0.6 million). In contrast, the gap between PIB estimates was highest for Punjab (highest CPR) and lowest in Bihar (lowest CPR) (Fig 3).

**Table 2:** State wise aggregates of BA and PIB by the two methods for the three years preceding the survey.

States	Total Districts	Number of Districts where TFR<2.1	Number of Districts where CPR>50	Total Births in last 3 years (1000)	BA (1000) (Method 1)	BA (1000) (Method 2)	PIB (Method 1)	PIB (Method 2)
Bigger States								
**Andhra Pradesh**	13	10	13	2,341	665	1,937	28.42	82.76
**Assam**	27	10	19	6,989	1,894	4,120	27.09	58.95
**Bihar**	38	0	0	10,100	2,036	2,670	20.16	26.44
**Chhattisgarh**	18	6	14	5,247	1,530	3,273	29.16	62.39
**Gujarat**	26	15	9	6,452	927	3,200	14.37	49.60
**Haryana**	21	14	17	5,520	1,386	4,083	25.11	73.97
**Jammu & Kashmir**	22	10	17	4,748	1,327	2,979	27.95	62.75
**Jharkhand**	24	4	3	5,735	1,323	2,409	23.07	42.00
**Karnataka**	30	26	20	7,202	1,194	4,043	16.57	56.13
**Kerala**	14	14	9	2,677	402	1,549	15.04	57.86
**Madhya Pradesh**	50	12	32	13,399	3,841	7,479	28.66	55.82
**Maharashtra**	35	24	32	8,110	2,091	6,124	25.78	75.51
**Odisha**	30	17	24	7,648	2,270	5,196	29.68	67.94
**Punjab**	20	20	20	5,168	1,501	4,734	29.05	91.60
**Rajasthan**	33	8	29	9,057	3,154	6,099	34.82	67.35
**Tamil Nadu**	32	32	17	6,008	723	3,250	12.03	54.10
**Telangana**	10	9	6	2,034	359	1,320	17.67	64.89
**Uttar Pradesh**	71	4	29	20,326	6,676	10,190	32.84	50.14
**Uttarakhand**	13	10	10	3,287	729	2,152	22.17	65.48
**West Bengal**	19	16	18	3,608	1,014	2,964	28.10	82.15
**Small States**								
**Arunanchal Pradesh**	16	10	3	3,342	21	59	0.64	1.75
**Goa**	2	2	0	246	2	33	0.85	13.56
**Himachal Pradesh**	12	10	9	2,879	44	159	1.54	5.54
**Manipur**	9	0	0	2,709	48	65	1.77	2.40
**Meghalaya**	7	2	0	1,896	36	58	1.91	3.04
**Mizoram**	8	2	0	2,016	47	87	2.33	4.29
**Nagaland**	11	2	0	2,588	33	62	1.27	2.41
**Sikkim**	4	4	3	932	39	134	4.16	14.40
**Tripura**	4	3	4	796	61	149	7.60	18.76
**Union Territories**								
**Andaman & Nicobar Island**	3	3	1	499	15	90	2.99	17.95
**Chandigarh**	1	1	1	114	33	103	28.61	89.81
**Dadara & Nagar Havelli**	1	0	0	191	56	75	29.49	38.97
**Daman & Diu**	2	2	0	253	3	51	1.21	20.29
**Lakshadweep**	1	1	0	213	4	63	1.83	29.33
**NCT of Delhi**	9	8	4	1,271	23	80	1.77	6.31
**Puducherry**	4	4	2	635	24	102	3.79	16.05
**India**	**640**	**315**	**365**	**156,238**	**38,184**	**87,933**	**24.44**	**56.28**

**Fig 4:**
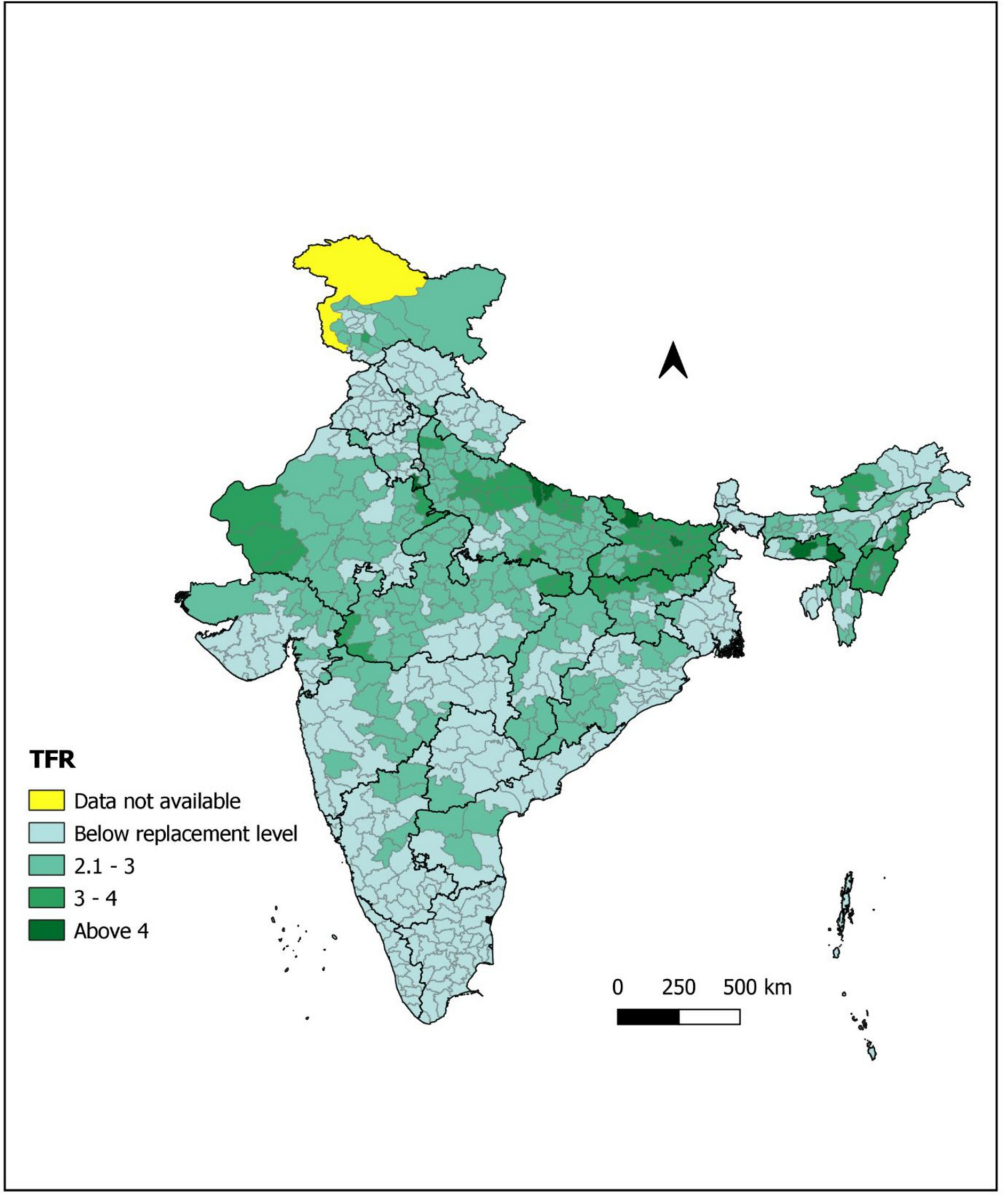
District wise distribution of observed Total Fertility Rate (TFR) based on three years reference period (Source: author’s calculations).

Fig 4 shows the district-wise distribution of observed TFR. District level TFR ranges between 0.99 and 5.67. Overall, still, nine districts (Mewat from Haryana; Jaintia Hills and West Khasi hills from Meghalaya; Bahraich and Shrawasti from Uttar Pradesh; Sheohar, Saharsa and Purba Champaran from Bihar) have TFR more than 4. Nearly, 14 percent districts have TFR above 3. The spread of these districts was limited to Rajasthan, Uttar Pradesh, Madhya Pradesh, Bihar, Jharkhand, Manipur, Meghalaya, Nagaland, Arunachal Pradesh and Jammu & Kashmir only. Around 50 percent (315 districts) have below replacement level fertility (2.1). Results from Table 2 shows that all the districts of Kerala, Punjab and Tamil Nadu among bigger states and all the districts of Goa and Sikkim among small states have achieved below-replacement fertility. Besides these, all the Union Territories except Dadara & Nagar Haveli and NCT of Delhi (83 percent) have achieved below replacement fertility level. Similarly, among other bigger states, Telangana (90 percent), Karnataka (87 percent), West Bengal (83 percent), Uttarakhand and Andhra Pradesh (both 77 percent) have already achieved below-replacement fertility. Bihar (Nil) followed by Uttar Pradesh (6 percent), Jharkhand (17 percent), Rajasthan and Madhya Pradesh (both 24 percent) were the poor performing states among bigger states for the achievement of below-replacement fertility level. Among other smaller states, 83 percent of districts of Himachal Pradesh, followed by Tripura (75 percent) and Arunanchal Pradesh (63 percent) have below-replacement fertility level. Manipur (Nil), Nagaland (18 percent), Mizoram (25 percent) and Meghalaya (29 percent) were very poor performing small states in achieving below replacement fertility level.

**Fig 5:**
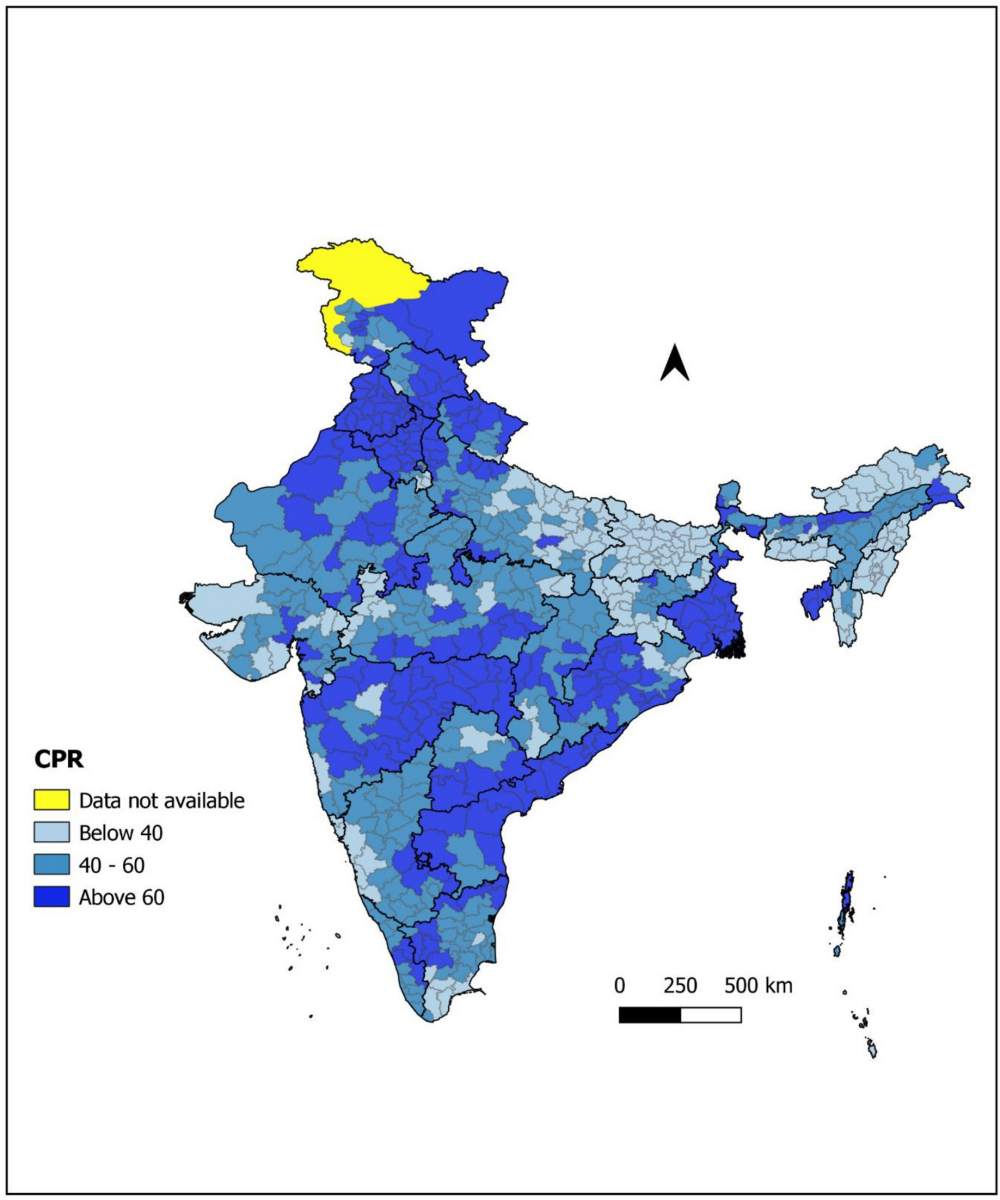
District wise distribution of Contraceptive Prevalence Rate (CPR) among currently married women of age 15–49 years (Source: author’s calculations).

Fig 5 shows district-wise distribution of CPR (for currently married women of age 15–49) among 640 districts of India. The range of CPR lied between 2.7 to 84.8 percent. Ten districts (Balrampur and Shrawasti from Uttar Pradesh; Pashchim Champaran, Purba Champaran, Saran (Chhapra), Gopalganj, Muzaffarpur and Siwan from Bihar; East Kameng and West Siang from Arunachal Pradesh) have less than 10 percent contraceptive prevalence. Nearly 6 percent (38 districts) have CPR below 20 percent. These districts spread over the Uttar Pradesh, Bihar, Madhya Pradesh, Jharkhand, Haryana, Arunachal Pradesh, Manipur, Meghalaya and Nagaland. However, no districts in Punjab, Rajasthan, Andhra Pradesh, Kerala, West Bengal and Tripura have CPR below 40 percent. The large patch of districts having TFR below 40 percent found in the middle and eastern Uttar Pradesh, Bihar, Jharkhand, some districts of Odisha that were along the border of Jharkhand and in northeastern states (Manipur, Meghalaya, Nagaland, Mizoram and Arunachal Pradesh). Apart from these, some were located in Southern Tamil Nadu, coastal districts of Karnataka, Goa, Gujarat and Madhya Pradesh. Only one or two such districts were situated in Haryana, Himachal Pradesh, Uttarakhand, Chhattisgarh, Telangana Sikkim and Assam. There were 30 districts where CPR was more than 75 percent, mostly located in Punjab followed by Haryana, West Bengal and Maharashtra.

It turns out from Table 2 that 365 districts out of 640, have more than 50 contraceptive prevalence rate. All the districts of Andhra Pradesh, Punjab, and Tripura among all states and Chandigarh among all union territory have more than 50 contraception prevalence rate. 95 percent districts of the West Bengal, 91 percent districts of Maharashtra and 88 percent districts of Rajasthan have more than 50 CPR. On the other side, no districts of Bihar, Manipur, Meghalaya, Mizoram and Nagaland among all the states and Dadara & Nagar Haveli, Daman & Diu and Lakshadweep among all the union territory have more than 50 contraceptive prevalence rate. Only 13 percent districts of Jharkhand, 19 percent districts of Arunachal Pradesh, 35 percent districts of the Gujarat and 41 percent districts of Uttar Pradesh have use of any contraception more than 50. Even in the NCT of Delhi, only 44 percent of districts have more than 50 CPR. Goa is the only state where all the districts were having below replacement fertility level, and CPR is also below 50.

**Fig 6:**
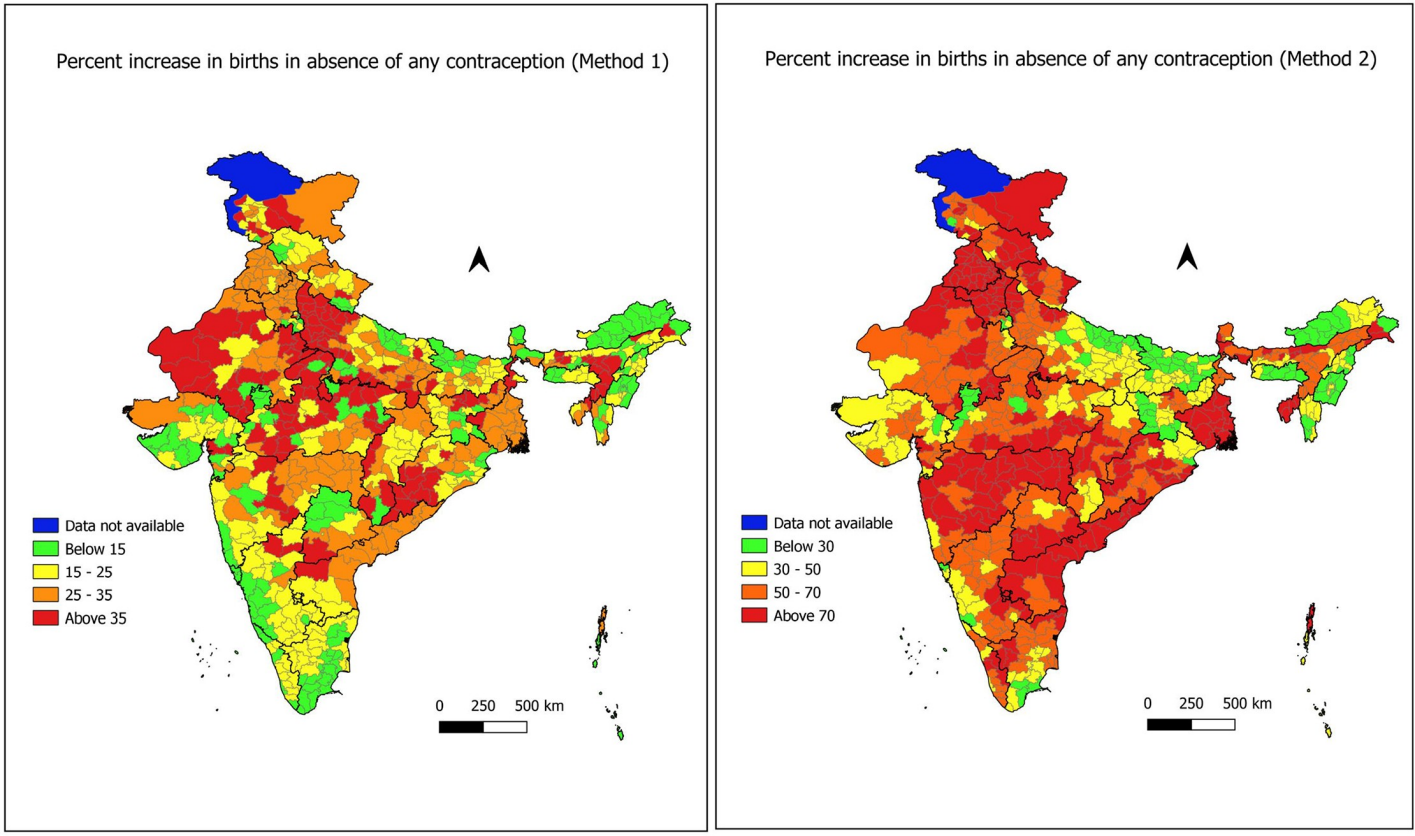
(a) District wise percentage increase in births in absence of contraception (PIB) obtained from Method 1 (Source: author’s calculations). (b) District wise percentage increase in births in absence of contraception (PIB) obtained from Method 2 (Source: author’s calculations).

Fig 6(A) shows the distribution of PIB estimates obtained from method 1 for Indian districts. Around 22 percent districts showed less than 15 percent growth in births in the absence of contraception. In other words, these districts exhibited less than 15 percent reduction in TFR due to use of contraception. These districts were located in all parts of the country except Punjab, Andhra Pradesh and Tripura states. Another 30 percent districts indicated 15 to 25 percent growth in births when no women would be using any contraception. A cluster of such districts found mainly in south India particularly in Kerala, Karnataka, Tamil Nadu. Very few districts (either 1 or 2) from Punjab, Haryana, West Bengal and Telangana found in this class. 27 percent districts predominantly situated in Punjab, Haryana, Rajasthan, West Bengal, Andhra Pradesh and Maharashtra showed 25 to 35 percent increase in births in the absence of contraception. Remaining 21 percent districts showed more than 35 percent reduction in births due to contraception. The cluster of these districts found in Rajasthan, Western Uttar Pradesh, Madhya Pradesh, Odisha and Assam.

Fig 6(B) portrays the distribution of estimates of PIB obtained through method 2 among districts of India. Nearly 14 percent districts predominantly found in Uttar Pradesh, Bihar, Jharkhand, Arunachal Pradesh, Meghalaya and Manipur exhibited less than 30 percent growth of births in the absence of all forms of contraception. Around 23 percent districts indicated 30 to 50 percent reduction in TFR due to contraception and these sets were found mostly in central India, Gujarat, Karnataka, Tamil Nadu and Arunachal Pradesh. Further, 32 percent districts showed 50 to 70 percent increase in births if no women would be using any form of contraception. Most of these districts located in Rajasthan, Madhya Pradesh, Western Uttar Pradesh, Karnataka, Telangana, Assam and Tamil Nadu. Rest districts were mostly situated in Punjab, Haryana, Himachal Pradesh, Maharashtra, Telangana, Andhra Pradesh, Tripura and West Bengal exhibited more than 70 percent reduction in TFR due to use of family planning methods. Some of the districts that fall in this category were situated in Uttarakhand, Uttar Pradesh, Madhya Pradesh, Chhattisgarh, Tamil Nadu and Kerala.

A comparison of two approaches using state-specific percentage increase in birth in the absence of any contraception (PIB) clearly showed that method 2 consistently giving a higher estimate than that of method 1 (Table 2). In most states, the estimate from method 2 was two or three times the estimate obtained from method 1. Punjab ranked the first among all the states and union territories with the highest percent increase in the births (92 percent) using method 2, while, using method 1, Rajasthan gained the highest percent increase in births (35 percent). However, Arunachal Pradesh ranked the lowest percentage increase in births among all the states and union territories by both the methods.

### Sensitivity analysis of BA and PIB estimates

**Fig 7:**
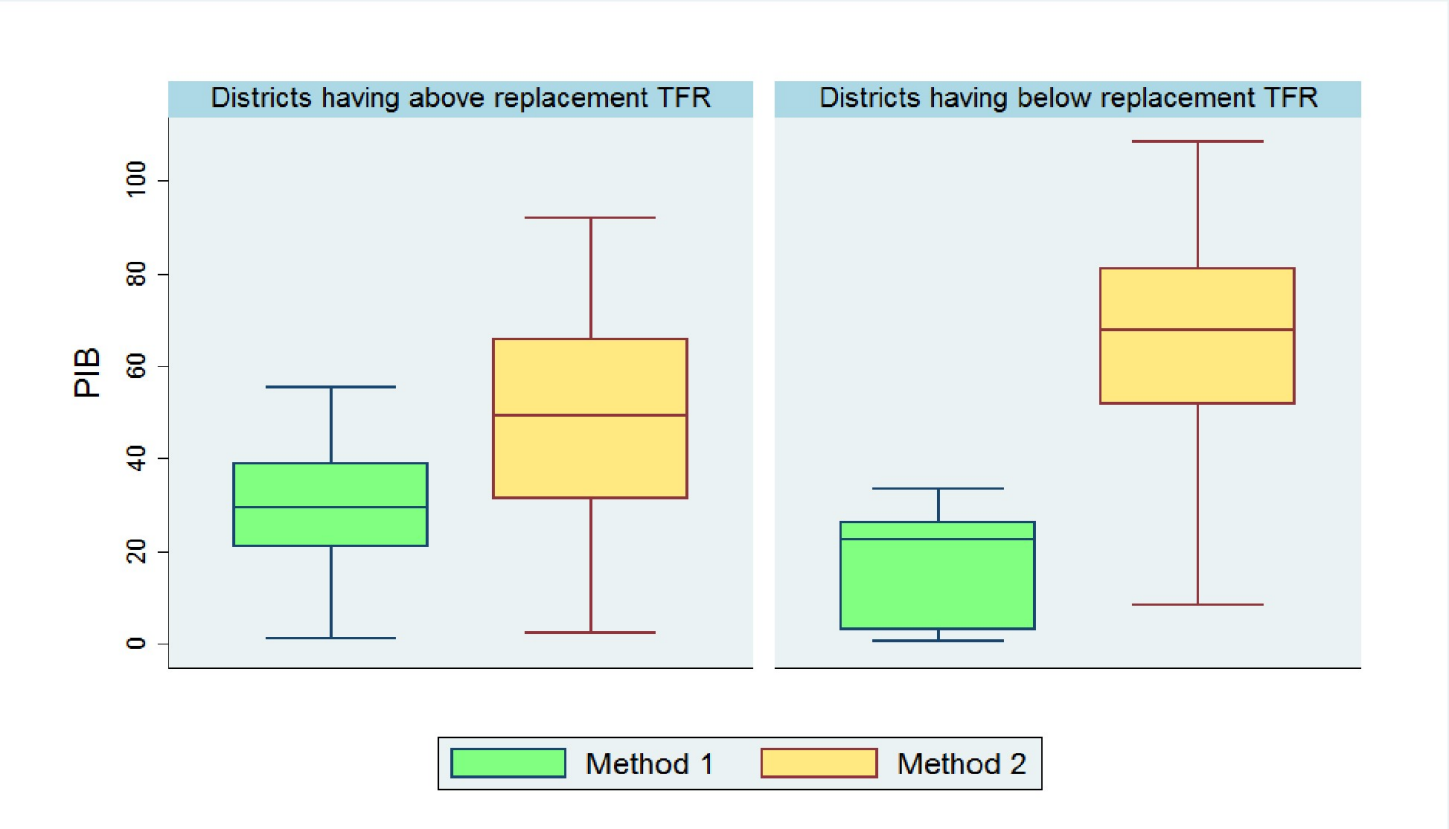
Box plots comparing the two estimates of PIB among 640 districts of India based on replacement level TFR criteria.

Fig 7 displays box plots of percentage increase in births in the absence of any contraception under both methods of estimation for all 640 districts. Here, the criteria for comparison was based on replacement level TFR (2.1). In general, we can say that method 2 was giving every time higher estimates than that of method 1. Analyzing the spread of lower (25th percentile) and upper (75th percentile) bound of percent increase in births for the districts having TFR above replacement level (total 325 districts), we found that, they were 21–39 percent for method 1 and 31–65 percent for method 2. Similarly, for the districts having below replacement TFR level (total 315 districts), the lower (25th percentile) and upper (75th percentile) bound of percent increase in births were 3–26 percent under method 1 and 52–81 percent under method 2. The distribution of estimates of percentage increase in births under both methods was symmetric for those districts having TFR more than replacement level. The median percentage increase in births for districts having above replacement TFR was 29 and 49 percent under method 1 and 2, respectively. However, the estimates of percentage increase in births for districts having below replacement level TFR were skewed towards left (Mean<Median) under both method 1 and 2. The extent of left skewness is greater for method 1 than that of method 2. The corresponding median estimates of percentage increase in births are 22 and 68 percent respectively.

The mean and standard deviation of the two estimates of birth averted due to contraception were considerably different from each other, so to see the variability of estimates in relation to the mean, we have also computed coefficients of variations (CV) of the two estimates of the number of birth averted due to contraception. For the districts having TFR above replacement level, the two CVs were almost same (CV of BA (method 1) is 6.5 percent, and CV of BA (method 2) was 6.6 percent) whereas, for districts having below replacement level TFR, the CV of BA under method 1 (8.5 percent) was higher than that of method 2 (6.2 percent). However, some districts from the group 1 & 3 and none from the group 2 & 4 under method 1, have TFR level less or equal to replacement level. Further, we found that the CV of BA under group 1 and 3 are 5.4 percent and 5.3 percent respectively, which was lesser than the CV of BA under method 2 (6.2 percent).

## Discussion & conclusion

To the best of our knowledge, no previous study gave a disaggregated analysis of the number of births averted as a result of the use of contraception using the most recent round of National Family Health Survey. In this study, two methods were applied and compared to estimate the number of births averted at district and state level. The period of reference of our estimates were 3 years before the survey date. According to the average of the estimate obtained by two methods, nearly 63 million births would have been averted by the use of contraception or 40 percent more than the number of births that occurred during that period. Our both approaches were different from the method used by earlier researchers [19, 20, 23] as other researchers either used linear regression or polynomial regression of TFR and CPR while our approaches are based on the linear relationship between logarithm transformation of TFR and CPR. The estimates obtained through our first Method 1 was always less than those obtained by Method 2. For most of the districts, the estimates from method 2 were between 1 to 3 times of the estimates obtained by method 1. On the other hand, for some districts where either count of exposed women or number of births or both were small, this ratio became 4 or more. The discrepancy between the estimates obtained from both methods was pronounced more in the districts where the relationship between TFR and CPR was not too strong. It means that when we have applied single robust regression (Method 2) to compute our estimates, it overestimated the births averted for those districts where TFR and CPR both are low (i.e. districts falling in group 1 of method 1). Meanwhile, it would also underestimate the births averted for the districts with high TFR and CPR. Alternatively, we can state that fitting a single equation to obtain estimates of BA may not be that helpful as districts of India were at various phase of fertility transition. Taking this heterogeneity into consideration, in method 1, we formed four homogenous groups and used four different robust regressions to estimate BA.

It was seen from the regression equations that if contraceptive prevalence fails to exist in society, i.e. CPR = 0, then by method 1, the TFRP for the districts falling in group 1, 2, 3, 4 would be 1.84, 3.66, 2.23 and 3.60 respectively whereas, by method 2, the same figure would be 3.39 children on an average per women. From these results we can infer that the districts in group 1 to 4 (under method 1) were very much heterogeneous in their TFR-CPR relationship. Therefore, method 1 is recommended over method 2 for getting the real picture of birth averted in Indian context. On the other hand, if all the women start using any method of contraception, TFR would be lowered down to 1.72, 1.83, 1.58 and 2.00 children on an average per women for the respective groups by method 1 and on an average 1.42 children per women by applying method 2.

Results from the sensitivity analysis conclude that for the districts where TFR is above the replacement level, the coefficient of variation for estimates of BA was more or less same while for the districts having below-replacement fertility, methods 1 will work better against method 2. Overall, these results also recommend to prefer method 1 over method 2. One can take the average of these two estimates of BA as final estimates of BA. Further, we figured out from taking a closer look at Figs 5 and 6(B) that there was a great resemblance between two figures. Results of method 2, suggests that the more the use of contraception, the more the percentage increase in births that would be avoided due to contraception. However, the results of method 1 were not concluding the same. By looking at Figs 4, 5 and 6(A), we found that there were some districts in Kerala, Tamil Nadu, Karnataka, Telangana, Maharashtra, Gujarat, Madhya Pradesh, Uttar Pradesh, Jharkhand, Himachal Pradesh and Delhi where the contraceptive prevalence is more than 40 percent and TFR is below replacement level. However, the percentage increase in births found to be lower than 15 percent. It indicates that there may be an influence of other factors like postponement of age at marriage, first birth, rise in divorce rates, more involvement of women in education and workforce, increasing rate of abortions, shortening the duration of breastfeeding or maybe decreasing utility of another child. These factors might appear with the change in the behaviour of society due to the influence of westernization and rapid urbanization. Now couples are willing to have a smaller family as compared to their desire in the past.

It is worth to mention that there is an enormous disparity in contraceptive use as well as in total fertility rate among districts which was also evident by survey report and previous literature. We have also spotted distinct geographical clustering of districts for both CPR, TFR and PIB estimates. Further we found that there is a considerable variation in the estimates of the number of births averted among different districts as well as states of India. This variation might be owing to difference in the contraceptive prevalence across districts or difference in the composition of contraceptives (effective method mix) used by the couples in those districts. For example, the range of CPR lies between 2.7 to 84.8 percent among Indian districts as per NFHS-4 [7]. The variation in births averted among district could be best explained by methods of contraception used. Due to different method mix, overall effectiveness of contraception varies. Districts/states where the prevalence of sterilization is high typically have lower fertility than district/states that have same contraceptive prevalence but lower use of long term methods. For instance, Andhra Pradesh and Punjab both have high modern contraceptive use (69.4 and 66.3 percent respectively) among currently married women of age 15–49 [7]. However, In Andhra Pradesh, 68.9 percent couples use permanent sterilization (Male/Female) and only 0.2 percent couples use condom/Nirodh while in Punjab, this fraction is 38.1 and 18.9 percent respectively. Since sterilization is assumed to be most effective method of contraception. Therefore, the overall effectiveness of CPR in Andhra Pradesh is higher than that of Punjab. Additionally, the number of averted births also depend on the size and age structure of the population of an area (districts/states) as the total number of births and exposed number of women in that area depend on those characteristics. Even if, the sample size of each district is chosen in such a way that it can provide an estimate at the district level, further validity of the estimates of BA for smaller district needs to be tested.

Previous reports of NFHS also shows that female sterilization is the most preferred method (36 percent prevalence, according to NFHS-4 [7]) of contraception in India. A significant fraction of women never uses any contraception before going for permanent sterilization. As per NFHS-3 & 4, more than 50 percent of the women undertook female sterilization before reaching 26 years [6, 7]. Although permanent sterilization is assumed to be very powerful for birth control, still if it is not adopted at desired parity, the objective of using contraception will not be achieved in real sense. At the same time, the prevalence of reversible methods of contraception is very low in our country. Therefore, the focus of the government should be shifted from the promotion of permanent methods to the long acting reversible methods of birth control.

After launching of *National Health Mission*, districts are made the programming, planning and financial entity. Now it depends on the government functionaries at the district level how well our health schemes improve the conditions of the districts. The added value of this study lies in the fact that it can spot the districts that are not performing well at the front of the utilization of various family planning methods for birth control. These results have enormous relevance for policymakers, government authorities, and health professionals. It will help them to formulate specific strategies and programs of intervention for the sexually active unmarried and married women of poor-performing districts. Our methods can also be used to produce the estimates of the number of births that would have been unplanned (either unintended or mistimed) that had not been prevented by practice of contraception. The results of this study provide a basis for further approximation of the risks of unsafe abortions and maternal deaths prevented by practice of contraception. Our study strengthened the previously established result that contraceptive practice has a strong impact on the fertility. Government of India has taken several new initiatives like *Mission Parivar Vikas*, home delivery of contraceptives by ASHA worker, emphasis on Postpartum Family Planning (PPFP), awareness regarding the new and reversible basket of choice for the contraceptives, etc., as part of National Family Welfare Programme [25]. The government must trace the functioning, monitoring and evaluation of all government interventions in these poor performing districts in the way as the government is monitoring the progress of ‘*Aspirational Districts*’ [26].

### Limitations of the study

Since our study is based on all methods of contraception (either traditional or modern), the separate estimates of births averted due to modern as well as traditional methods of contraception are not worked out. Further, our estimates will underestimate the count of birth averted in those districts where couples are using effective method mix, and it will overestimate the same where method mix is less effective. For getting insights into this variation in the number of birth averted due to various contraceptives, there is excellent scope for more in-depth analysis. Another limitation of our study is that we assumed that the TFR and CPR relationship is same for all the women (unmarried and married). In fact unmarried women practices contraception less frequently than that of married women. We have not used CPR (all women) because there is no utilization of using contraception for the women outside union as they are not contributing into fertility.

### Source of Indian digital map

All the maps were made in QGIS 3.10 software by the authors themselves based on authors’ calculations specifically for this manuscript. The district level shape file (digital map) of India was obtained from GitHub at https://github.com/datameet/maps/tree/master/Districts. The digital map has been used under the Creative Commons Attribution 2.5 India license. The shape file was created using the administrative atlas of Census 2011, India. And the map was projected in WGS 1984 UTM zone 43N.

## Supporting information

S1 Table District wise TFR and 95% CI, CPR, total births, estimates of BA and PIB by the two methods for the three years preceding the survey. (DOCX)
