# Supplementary material for: Disaggregated analysis of birth averted due to family planning use in India: An evidence from NFHS-4 (2015–16)
Source: PLoS One. 2020 Sep 23;15(9):e0239376. doi: 10.1371/journal.pone.0239376 (PMC7511029; doi:10.1371/journal.pone.0239376)
Supplement: S1 Table — (DOCX) [file pone.0239376.s001.docx]

**S1 Table: District wise TFR and 95% CI, CPR, Total births, estimates of BA and PIB by the two methods for the three years preceding the survey**

| **Sr.no.** | **District** | **Total birth in last 3 years (1000)** | **CPR** | **Lower TFR actual** | **TFR actual** | **Upper TFR actual** | **Method 1** | | | **Method 2** | | |
| --- | --- | --- | --- | --- | --- | --- | --- | --- | --- | --- | --- | --- |
|  |  |  |  |  |  |  | **TFR potential** | **BA (1000)** | **PIB** | **TFR potential** | **BA (1000)** | **PIB** |
|  | **Andaman & Nicobar Island** | | |  |  |  |  |  |  |  |  |  |
| 1 | Nicobar | 154 | 38.00 | 1.43 | 1.70 | 1.97 | 1.740 | 4 | 2.35 | 2.362 | 60 | 38.97 |
| 2 | North & Middle Andaman | 138 | 66.90 | 1.19 | 1.46 | 1.73 | 1.833 | 35 | 25.54 | 2.606 | 109 | 78.49 |
| 3 | South Andaman | 206 | 45.60 | 1.16 | 1.41 | 1.66 | 1.450 | 6 | 2.82 | 2.093 | 100 | 48.42 |
|  | **Andhra Pradesh** | |  |  |  |  |  |  |  |  |  |  |
| 4 | Anantapur | 202 | 65.30 | 1.60 | 1.91 | 2.22 | 2.385 | 50 | 24.86 | 3.362 | 153 | 76.03 |
| 5 | Chittoor | 149 | 59.70 | 1.67 | 1.99 | 2.31 | 2.438 | 33 | 22.50 | 3.337 | 101 | 67.70 |
| 6 | East Godavari | 129 | 72.20 | 1.30 | 1.58 | 1.86 | 2.020 | 36 | 27.82 | 2.953 | 112 | 86.87 |
| 7 | Guntur | 334 | 74.00 | 1.50 | 1.81 | 2.12 | 2.328 | 96 | 28.61 | 3.436 | 300 | 89.81 |
| 8 | Krishna | 187 | 74.80 | 1.19 | 1.45 | 1.72 | 1.870 | 54 | 28.96 | 2.771 | 170 | 91.13 |
| 9 | Kurnool | 182 | 65.90 | 1.91 | 2.21 | 2.51 | 3.260 | 86 | 47.52 | 3.911 | 140 | 76.95 |
| 10 | Prakasam | 274 | 70.90 | 1.80 | 2.12 | 2.45 | 2.698 | 75 | 27.26 | 3.917 | 232 | 84.78 |
| 11 | Sri Potti Sriramulu Nellore | 152 | 71.00 | 1.64 | 1.94 | 2.25 | 2.470 | 41 | 27.30 | 3.588 | 129 | 84.94 |
| 12 | Srikakulam | 143 | 67.70 | 1.61 | 1.91 | 2.22 | 2.404 | 37 | 25.88 | 3.433 | 114 | 79.73 |
| 13 | Visakhapatnam | 146 | 69.20 | 1.26 | 1.55 | 1.84 | 1.961 | 39 | 26.53 | 2.822 | 120 | 82.08 |
| 14 | Vizianagaram | 104 | 71.60 | 1.52 | 1.82 | 2.11 | 2.322 | 29 | 27.56 | 3.383 | 89 | 85.90 |
| 15 | West Godavari | 178 | 76.70 | 1.33 | 1.64 | 1.94 | 2.129 | 53 | 29.79 | 3.186 | 168 | 94.30 |
| 16 | Y.S.R(Cuddapah) | 163 | 59.40 | 1.79 | 2.12 | 2.45 | 2.594 | 37 | 22.38 | 3.546 | 110 | 67.26 |
|  | **Arunanchal Pradesh** | |  |  |  |  |  |  |  |  |  |  |
| 17 | Anjaw | 139 | 24.50 | 1.43 | 1.72 | 2.02 | 1.746 | 2 | 1.51 | 2.127 | 33 | 23.64 |
| 18 | Changlang | 174 | 62.60 | 1.63 | 1.92 | 2.21 | 2.375 | 41 | 23.72 | 3.302 | 125 | 71.96 |
| 19 | Dibang Valley | 131 | 44.00 | 1.52 | 1.84 | 2.16 | 1.890 | 4 | 2.72 | 2.693 | 61 | 46.38 |
| 20 | East Kameng | 244 | 5.20 | 3.06 | 3.51 | 3.95 | 3.636 | 9 | 3.60 | 3.672 | 11 | 4.61 |
| 21 | East Siang | 301 | 32.50 | 0.99 | 1.22 | 1.46 | 1.244 | 6 | 2.00 | 1.617 | 98 | 32.50 |
| 22 | Kurung Kumey | 249 | 12.90 | 2.76 | 3.16 | 3.56 | 3.450 | 23 | 9.17 | 3.533 | 29 | 11.82 |
| 23 | Lohit | 195 | 66.00 | 2.14 | 2.45 | 2.76 | 3.616 | 93 | 47.61 | 4.339 | 150 | 77.10 |
| 24 | Lower Dibang Valley | 119 | 31.00 | 1.70 | 2.00 | 2.31 | 2.038 | 2 | 1.91 | 2.616 | 36 | 30.79 |
| 25 | Lower Subansiri | 286 | 10.60 | 1.46 | 1.79 | 2.12 | 1.802 | 2 | 0.65 | 1.962 | 28 | 9.61 |
| 26 | Papumpare | 205 | 12.80 | 1.70 | 1.91 | 2.11 | 1.925 | 2 | 0.78 | 2.134 | 24 | 11.72 |
| 27 | Tawang | 206 | 22.30 | 2.14 | 2.55 | 2.95 | 2.968 | 34 | 16.37 | 3.093 | 44 | 21.30 |
| 28 | Tirap | 160 | 57.80 | 1.38 | 1.63 | 1.88 | 1.984 | 35 | 21.72 | 2.689 | 104 | 64.96 |
| 29 | Upper Siang | 188 | 38.90 | 0.90 | 1.14 | 1.39 | 1.167 | 5 | 2.40 | 1.597 | 75 | 40.06 |
| 30 | Upper Subansiri | 194 | 17.90 | 2.01 | 2.35 | 2.69 | 2.654 | 25 | 12.94 | 2.744 | 33 | 16.77 |
| 31 | West Kameng | 422 | 18.90 | 2.16 | 2.55 | 2.94 | 2.900 | 58 | 13.71 | 3.003 | 75 | 17.78 |
| 32 | West Siang | 129 | 9.30 | 1.44 | 1.76 | 2.06 | 1.770 | 1 | 0.57 | 1.908 | 11 | 8.39 |
|  | **Assam** |  |  |  |  |  |  |  |  |  |  |  |
| 33 | Baksa | 227 | 58.10 | 1.76 | 2.02 | 2.29 | 2.461 | 50 | 21.84 | 3.341 | 149 | 65.39 |
| 34 | Barpeta | 251 | 56.70 | 2.07 | 2.36 | 2.66 | 3.298 | 100 | 39.73 | 3.856 | 159 | 63.40 |
| 35 | Bongaigaon | 225 | 59.40 | 1.92 | 2.21 | 2.50 | 3.138 | 95 | 41.97 | 3.697 | 152 | 67.26 |
| 36 | Cachar | 225 | 54.40 | 1.99 | 2.29 | 2.59 | 3.157 | 85 | 37.85 | 3.668 | 135 | 60.18 |
| 37 | Chirang | 249 | 62.60 | 2.22 | 2.53 | 2.85 | 3.660 | 111 | 44.68 | 4.351 | 179 | 71.96 |
| 38 | Darrang | 247 | 65.70 | 2.07 | 2.38 | 2.68 | 3.507 | 117 | 47.35 | 4.204 | 189 | 76.64 |
| 39 | Dhemaji | 263 | 54.80 | 2.01 | 2.29 | 2.57 | 3.164 | 100 | 38.17 | 3.681 | 160 | 60.73 |
| 40 | Dhubri | 281 | 34.20 | 2.47 | 2.80 | 3.13 | 3.533 | 74 | 26.18 | 3.765 | 97 | 34.47 |
| 41 | Dibrugarh | 186 | 53.80 | 1.46 | 1.71 | 1.96 | 2.053 | 37 | 20.07 | 2.725 | 110 | 59.35 |
| 42 | Dima Hasao | 246 | 48.70 | 2.26 | 2.59 | 2.92 | 3.607 | 97 | 39.26 | 3.949 | 129 | 52.46 |
| 43 | Goalpara | 338 | 43.70 | 2.48 | 2.81 | 3.14 | 3.782 | 117 | 34.60 | 4.103 | 156 | 46.00 |
| 44 | Golaghat | 258 | 46.00 | 1.47 | 1.73 | 1.98 | 1.779 | 7 | 2.85 | 2.577 | 126 | 48.94 |
| 45 | Hailakandi | 507 | 54.20 | 2.31 | 2.64 | 2.96 | 3.635 | 191 | 37.68 | 4.221 | 304 | 59.90 |
| 46 | Jorhat | 545 | 53.00 | 1.33 | 1.57 | 1.80 | 1.880 | 108 | 19.75 | 2.484 | 318 | 58.25 |
| 47 | Kamrup | 201 | 35.90 | 1.50 | 1.74 | 1.99 | 1.779 | 4 | 2.21 | 2.374 | 73 | 36.46 |
| 48 | Kamrup Metropolitan | 195 | 46.30 | 1.32 | 1.59 | 1.86 | 1.636 | 6 | 2.86 | 2.374 | 96 | 49.33 |
| 49 | Karbi Anglong | 329 | 53.30 | 1.95 | 2.25 | 2.55 | 3.081 | 122 | 36.95 | 3.570 | 193 | 58.66 |
| 50 | Karimganj | 238 | 52.10 | 2.47 | 2.78 | 3.09 | 3.780 | 86 | 35.99 | 4.365 | 136 | 57.02 |
| 51 | Kokrajhar | 159 | 56.70 | 1.89 | 2.18 | 2.48 | 2.644 | 34 | 21.26 | 3.562 | 101 | 63.40 |
| 52 | Lakhimpur | 399 | 49.10 | 1.87 | 2.16 | 2.44 | 2.226 | 12 | 3.04 | 3.305 | 211 | 52.99 |
| 53 | Morigaon | 154 | 51.10 | 2.64 | 2.97 | 3.31 | 4.015 | 54 | 35.19 | 4.623 | 86 | 55.66 |
| 54 | Nagaon | 137 | 53.70 | 2.49 | 2.83 | 3.17 | 3.885 | 51 | 37.28 | 4.506 | 81 | 59.21 |
| 55 | Nalbari | 139 | 63.20 | 1.63 | 1.89 | 2.15 | 2.343 | 33 | 23.97 | 3.267 | 101 | 72.86 |
| 56 | Sivasagar | 139 | 50.00 | 1.62 | 1.89 | 2.16 | 1.949 | 4 | 3.10 | 2.914 | 75 | 54.19 |
| 57 | Sonitpur | 116 | 64.10 | 1.45 | 1.70 | 1.95 | 2.114 | 28 | 24.35 | 2.962 | 86 | 74.21 |
| 58 | Tinsukia | 222 | 56.10 | 1.70 | 1.96 | 2.22 | 2.372 | 47 | 21.01 | 3.186 | 139 | 62.55 |
| 59 | Udalguri | 513 | 63.90 | 1.83 | 2.12 | 2.40 | 2.634 | 125 | 24.27 | 3.687 | 379 | 73.91 |
|  | **Bihar** |  |  |  |  |  |  |  |  |  |  |  |
| 60 | Araria | 446 | 29.90 | 3.56 | 3.93 | 4.30 | 4.816 | 100 | 22.55 | 5.092 | 132 | 29.55 |
| 61 | Arwal | 399 | 29.00 | 2.94 | 3.26 | 3.59 | 3.971 | 87 | 21.80 | 4.191 | 114 | 28.55 |
| 62 | Aurangabad | 191 | 32.80 | 2.36 | 2.66 | 2.97 | 3.325 | 48 | 24.99 | 3.534 | 63 | 32.85 |
| 63 | Banka | 378 | 26.90 | 3.01 | 3.36 | 3.70 | 4.034 | 76 | 20.07 | 4.241 | 99 | 26.23 |
| 64 | Begusarai | 396 | 35.20 | 2.95 | 3.28 | 3.62 | 4.167 | 107 | 27.04 | 4.449 | 141 | 35.64 |
| 65 | Bhagalpur | 417 | 24.60 | 3.11 | 3.45 | 3.79 | 4.078 | 76 | 18.21 | 4.269 | 99 | 23.74 |
| 66 | Bhojpur | 368 | 27.70 | 2.53 | 2.82 | 3.12 | 3.404 | 76 | 20.73 | 3.585 | 100 | 27.11 |
| 67 | Buxar | 371 | 35.00 | 2.62 | 2.92 | 3.22 | 3.705 | 100 | 26.87 | 3.954 | 131 | 35.41 |
| 68 | Darbhanga | 414 | 17.40 | 3.36 | 3.73 | 4.10 | 4.199 | 52 | 12.56 | 4.337 | 67 | 16.26 |
| 69 | Gaya | 637 | 35.70 | 3.04 | 3.35 | 3.66 | 4.270 | 175 | 27.48 | 4.564 | 231 | 36.23 |
| 70 | Gopalganj | 158 | 9.00 | 2.27 | 2.55 | 2.83 | 2.711 | 10 | 6.31 | 2.757 | 13 | 8.11 |
| 71 | Jamui | 345 | 23.70 | 3.30 | 3.65 | 4.00 | 4.288 | 60 | 17.49 | 4.482 | 79 | 22.78 |
| 72 | Jehanabad | 335 | 34.90 | 2.63 | 2.96 | 3.29 | 3.753 | 90 | 26.78 | 4.005 | 118 | 35.29 |
| 73 | Kaimur | 197 | 34.10 | 3.08 | 3.43 | 3.77 | 4.325 | 51 | 26.10 | 4.608 | 68 | 34.35 |
| 74 | Katihar | 219 | 26.60 | 3.26 | 3.65 | 4.05 | 4.374 | 43 | 19.83 | 4.596 | 57 | 25.90 |
| 75 | Khagaria | 226 | 28.30 | 3.53 | 3.89 | 4.26 | 4.715 | 48 | 21.22 | 4.970 | 63 | 27.77 |
| 76 | Kishanganj | 258 | 12.20 | 3.41 | 3.78 | 4.16 | 4.107 | 22 | 8.65 | 4.201 | 29 | 11.14 |
| 77 | Lakhisarai | 189 | 34.70 | 3.03 | 3.36 | 3.70 | 4.254 | 50 | 26.61 | 4.538 | 66 | 35.05 |
| 78 | Madhepura | 381 | 23.70 | 3.56 | 3.93 | 4.30 | 4.617 | 67 | 17.49 | 4.825 | 87 | 22.78 |
| 79 | Madhubani | 124 | 16.50 | 3.09 | 3.44 | 3.79 | 3.848 | 15 | 11.87 | 3.968 | 19 | 15.36 |
| 80 | Munger | 211 | 35.40 | 2.78 | 3.13 | 3.48 | 3.982 | 58 | 27.22 | 4.253 | 76 | 35.87 |
| 81 | Muzaffarpur | 152 | 9.20 | 2.80 | 3.15 | 3.50 | 3.353 | 10 | 6.46 | 3.411 | 13 | 8.29 |
| 82 | Nalanda | 205 | 30.50 | 2.85 | 3.21 | 3.56 | 3.950 | 47 | 23.05 | 4.180 | 62 | 30.23 |
| 83 | Nawada | 170 | 30.60 | 2.73 | 3.06 | 3.38 | 3.768 | 39 | 23.13 | 3.988 | 52 | 30.34 |
| 84 | Pashchim Champaran | 267 | 4.00 | 3.50 | 3.91 | 4.33 | 4.018 | 7 | 2.76 | 4.048 | 9 | 3.52 |
| 85 | Patna | 254 | 39.40 | 2.48 | 2.68 | 2.89 | 3.503 | 78 | 30.72 | 3.770 | 103 | 40.66 |
| 86 | Purba Champaran | 159 | 5.50 | 3.76 | 4.16 | 4.57 | 4.319 | 6 | 3.81 | 4.363 | 8 | 4.88 |
| 87 | Purnia | 236 | 31.60 | 3.53 | 3.91 | 4.29 | 4.847 | 57 | 23.97 | 5.141 | 74 | 31.48 |
| 88 | Rohtas | 171 | 44.80 | 2.59 | 2.88 | 3.18 | 3.906 | 61 | 35.61 | 4.245 | 81 | 47.40 |
| 89 | Saharsa | 333 | 29.20 | 3.84 | 4.24 | 4.64 | 5.171 | 73 | 21.96 | 5.460 | 96 | 28.77 |
| 90 | Samastipur | 179 | 13.00 | 3.33 | 3.72 | 4.11 | 4.064 | 17 | 9.24 | 4.163 | 21 | 11.92 |
| 91 | Saran | 218 | 8.70 | 2.91 | 3.26 | 3.61 | 3.459 | 13 | 6.09 | 3.515 | 17 | 7.83 |
| 92 | Sheikhpura | 310 | 32.60 | 3.27 | 3.62 | 3.96 | 4.518 | 77 | 24.82 | 4.801 | 101 | 32.62 |
| 93 | Sheohar | 128 | 18.70 | 3.85 | 4.27 | 4.69 | 4.849 | 17 | 13.56 | 5.021 | 23 | 17.58 |
| 94 | Sitamarhi | 161 | 33.80 | 3.36 | 3.73 | 4.11 | 4.694 | 42 | 25.84 | 4.998 | 55 | 34.01 |
| 95 | Siwan | 185 | 9.80 | 2.48 | 2.77 | 3.07 | 2.961 | 13 | 6.89 | 3.015 | 16 | 8.86 |
| 96 | Supaul | 158 | 33.70 | 3.63 | 3.99 | 4.35 | 5.018 | 41 | 25.75 | 5.342 | 53 | 33.89 |
| 97 | Vaishali | 155 | 24.00 | 2.89 | 3.21 | 3.52 | 3.779 | 27 | 17.73 | 3.952 | 36 | 23.10 |
|  | **Chandigarh** | |  |  |  |  |  |  |  |  |  |  |
| 98 | Chandigarh | 114 | 74.00 | 1.28 | 1.57 | 1.86 | 2.019 | 33 | 28.61 | 2.980 | 103 | 89.81 |
|  | **Chhattisgarh** | |  |  |  |  |  |  |  |  |  |  |
| 99 | Bastar | 278 | 45.80 | 2.12 | 2.40 | 2.69 | 3.277 | 102 | 36.54 | 3.568 | 135 | 48.68 |
| 100 | Bijapur | 186 | 51.40 | 2.29 | 2.58 | 2.86 | 3.494 | 66 | 35.43 | 4.027 | 104 | 56.07 |
| 101 | Bilaspur | 326 | 64.60 | 2.41 | 2.71 | 3.01 | 3.967 | 151 | 46.40 | 4.742 | 244 | 74.97 |
| 102 | Dakshin Bastar Dantewada | 255 | 39.40 | 1.93 | 2.20 | 2.48 | 2.254 | 6 | 2.43 | 3.095 | 103 | 40.66 |
| 103 | Dhamtari | 200 | 72.00 | 1.46 | 1.70 | 1.93 | 2.172 | 56 | 27.74 | 3.171 | 173 | 86.55 |
| 104 | Durg | 413 | 66.60 | 1.67 | 1.85 | 2.03 | 2.320 | 105 | 25.41 | 3.293 | 323 | 78.03 |
| 105 | Janjgir Champa | 449 | 61.70 | 1.44 | 1.69 | 1.95 | 2.084 | 105 | 23.34 | 2.884 | 317 | 70.63 |
| 106 | Jashpur | 185 | 50.10 | 2.25 | 2.57 | 2.90 | 3.454 | 64 | 34.39 | 3.966 | 100 | 54.32 |
| 107 | Kabirdham | 337 | 58.10 | 2.07 | 2.36 | 2.65 | 3.325 | 138 | 40.89 | 3.903 | 220 | 65.39 |
| 108 | Korba | 501 | 55.50 | 2.00 | 2.19 | 2.39 | 2.645 | 104 | 20.77 | 3.541 | 309 | 61.71 |
| 109 | Korea | 374 | 50.60 | 2.13 | 2.34 | 2.55 | 3.154 | 130 | 34.79 | 3.627 | 206 | 54.99 |
| 110 | Mahasamund | 204 | 65.50 | 1.87 | 2.15 | 2.43 | 2.686 | 51 | 24.94 | 3.791 | 155 | 76.34 |
| 111 | Narayanpur | 202 | 40.00 | 2.30 | 2.57 | 2.85 | 3.373 | 63 | 31.26 | 3.634 | 84 | 41.40 |
| 112 | Raigarh | 155 | 52.30 | 1.72 | 2.01 | 2.29 | 2.401 | 30 | 19.46 | 3.162 | 89 | 57.29 |
| 113 | Raipur | 284 | 57.50 | 1.88 | 2.07 | 2.26 | 2.517 | 61 | 21.59 | 3.406 | 184 | 64.53 |
| 114 | Rajnandgaon | 277 | 64.30 | 2.39 | 2.67 | 2.95 | 3.902 | 128 | 46.14 | 4.660 | 207 | 74.51 |
| 115 | Surguja | 350 | 41.40 | 2.26 | 2.59 | 2.92 | 3.432 | 114 | 32.51 | 3.707 | 151 | 43.12 |
| 116 | Uttar Bastar Kanker | 269 | 55.90 | 1.54 | 1.80 | 2.05 | 2.177 | 56 | 20.93 | 2.921 | 167 | 62.27 |
|  | **Dadara & Nagar Havelli** | | |  |  |  |  |  |  |  |  |  |
| 117 | Dadra Nagar Haweli | 191 | 38.00 | 1.98 | 2.32 | 2.67 | 3.004 | 56 | 29.49 | 3.224 | 75 | 38.97 |
|  | **Daman & Diu** | |  |  |  |  |  |  |  |  |  |  |
| 118 | Daman | 103 | 29.00 | 1.29 | 1.60 | 1.91 | 1.629 | 2 | 1.78 | 2.057 | 29 | 28.55 |
| 119 | Diu | 150 | 45.90 | 1.68 | 2.01 | 2.33 | 2.067 | 4 | 2.84 | 2.991 | 73 | 48.81 |
|  | **Goa** |  |  |  |  |  |  |  |  |  |  |  |
| 120 | North Goa | 128 | 20.90 | 1.33 | 1.62 | 1.90 | 1.641 | 2 | 1.28 | 1.941 | 25 | 19.84 |
| 121 | South Goa | 119 | 34.60 | 1.41 | 1.73 | 2.04 | 1.767 | 3 | 2.13 | 2.334 | 41 | 34.94 |
|  | **Gujarat** |  |  |  |  |  |  |  |  |  |  |  |
| 122 | Ahmadabad | 136 | 60.20 | 1.61 | 1.94 | 2.27 | 2.381 | 31 | 22.71 | 3.267 | 93 | 68.43 |
| 123 | Amreli | 133 | 37.60 | 1.39 | 1.68 | 1.97 | 1.719 | 3 | 2.32 | 2.327 | 51 | 38.49 |
| 124 | Anand | 173 | 51.50 | 1.78 | 2.09 | 2.41 | 2.490 | 33 | 19.14 | 3.265 | 97 | 56.20 |
| 125 | Banas Kantha | 247 | 42.40 | 2.37 | 2.72 | 3.07 | 3.629 | 83 | 33.42 | 3.927 | 110 | 44.37 |
| 126 | Bharuch | 164 | 68.20 | 1.89 | 2.23 | 2.57 | 3.335 | 81 | 49.54 | 4.025 | 132 | 80.51 |
| 127 | Bhavnagar | 162 | 34.40 | 1.52 | 1.80 | 2.08 | 1.838 | 3 | 2.12 | 2.425 | 56 | 34.70 |
| 128 | Dohad | 216 | 31.00 | 2.42 | 2.80 | 3.17 | 3.457 | 51 | 23.47 | 3.662 | 67 | 30.79 |
| 129 | Gandhinagar | 167 | 59.70 | 1.84 | 2.17 | 2.50 | 2.658 | 38 | 22.50 | 3.639 | 113 | 67.70 |
| 130 | Jamnagar | 155 | 35.80 | 1.46 | 1.73 | 2.00 | 1.768 | 3 | 2.21 | 2.359 | 56 | 36.35 |
| 131 | Junagadh | 174 | 55.90 | 1.55 | 1.84 | 2.12 | 2.225 | 37 | 20.93 | 2.986 | 109 | 62.27 |
| 132 | Kachchh | 270 | 37.70 | 1.98 | 2.31 | 2.63 | 2.985 | 79 | 29.22 | 3.202 | 104 | 38.61 |
| 133 | Kheda | 325 | 32.70 | 2.03 | 2.36 | 2.69 | 2.948 | 81 | 24.90 | 3.133 | 106 | 32.73 |
| 134 | Mahesana | 748 | 49.00 | 1.87 | 2.20 | 2.53 | 2.267 | 23 | 3.03 | 3.363 | 395 | 52.86 |
| 135 | Narmada | 203 | 55.00 | 1.97 | 2.29 | 2.61 | 3.168 | 78 | 38.33 | 3.687 | 124 | 61.01 |
| 136 | Navsari | 356 | 68.70 | 1.48 | 1.78 | 2.09 | 2.248 | 94 | 26.31 | 3.227 | 290 | 81.29 |
| 137 | Panchmahal | 158 | 24.00 | 1.98 | 2.30 | 2.62 | 2.708 | 28 | 17.73 | 2.831 | 37 | 23.10 |
| 138 | Patan | 185 | 41.90 | 1.66 | 1.95 | 2.24 | 2.000 | 5 | 2.59 | 2.803 | 81 | 43.74 |
| 139 | Porbandar | 163 | 35.50 | 1.45 | 1.71 | 1.98 | 1.747 | 4 | 2.19 | 2.325 | 59 | 35.99 |
| 140 | Rajkot | 360 | 44.50 | 1.42 | 1.69 | 1.97 | 1.737 | 10 | 2.75 | 2.485 | 169 | 47.02 |
| 141 | Sabarkantha | 280 | 46.50 | 1.86 | 2.18 | 2.50 | 2.243 | 8 | 2.88 | 3.261 | 139 | 49.58 |
| 142 | Surat | 168 | 48.90 | 1.49 | 1.77 | 2.04 | 1.824 | 5 | 3.03 | 2.703 | 89 | 52.73 |
| 143 | Suredranagar | 243 | 56.90 | 1.66 | 1.96 | 2.26 | 2.378 | 52 | 21.34 | 3.208 | 155 | 63.68 |
| 144 | Tapi | 172 | 51.30 | 1.35 | 1.62 | 1.88 | 1.929 | 33 | 19.06 | 2.526 | 96 | 55.93 |
| 145 | The Dangs | 140 | 38.60 | 2.58 | 2.93 | 3.29 | 3.809 | 42 | 30.02 | 4.093 | 55 | 39.69 |
| 146 | Vadodara | 419 | 46.30 | 1.77 | 2.09 | 2.42 | 2.150 | 12 | 2.86 | 3.121 | 207 | 49.33 |
| 147 | Valsad | 532 | 38.40 | 1.60 | 1.90 | 2.20 | 1.945 | 13 | 2.37 | 2.650 | 210 | 39.45 |
|  | **Haryana** |  |  |  |  |  |  |  |  |  |  |  |
| 148 | Ambala | 186 | 77.60 | 1.27 | 1.49 | 1.71 | 1.940 | 56 | 30.19 | 2.918 | 179 | 95.82 |
| 149 | Bhiwani | 244 | 73.50 | 1.84 | 2.11 | 2.37 | 2.709 | 69 | 28.39 | 3.988 | 217 | 88.99 |
| 150 | Faridabad | 277 | 28.30 | 1.86 | 2.15 | 2.45 | 2.187 | 5 | 1.74 | 2.747 | 77 | 27.77 |
| 151 | Fatehabad | 347 | 76.90 | 1.66 | 1.91 | 2.15 | 2.481 | 104 | 29.88 | 3.718 | 328 | 94.63 |
| 152 | Gurgaon | 160 | 51.10 | 1.66 | 1.93 | 2.20 | 2.296 | 30 | 18.97 | 3.004 | 89 | 55.66 |
| 153 | Hisar | 196 | 72.60 | 1.80 | 2.08 | 2.35 | 2.662 | 55 | 28.00 | 3.900 | 172 | 87.52 |
| 154 | Jhajjar | 356 | 74.40 | 1.58 | 1.83 | 2.10 | 2.357 | 103 | 28.78 | 3.486 | 322 | 90.47 |
| 155 | Jind | 239 | 76.60 | 1.69 | 1.94 | 2.19 | 2.517 | 71 | 29.75 | 3.766 | 225 | 94.13 |
| 156 | Kaithal | 388 | 76.00 | 1.82 | 2.10 | 2.37 | 2.719 | 114 | 29.49 | 4.056 | 361 | 93.12 |
| 157 | Karnal | 107 | 75.40 | 1.88 | 2.17 | 2.46 | 2.804 | 31 | 29.22 | 4.169 | 98 | 92.12 |
| 158 | Kurushektra | 358 | 74.00 | 1.36 | 1.60 | 1.83 | 2.058 | 102 | 28.61 | 3.037 | 322 | 89.81 |
| 159 | Mahendragarh | 170 | 70.80 | 1.42 | 1.66 | 1.90 | 2.112 | 46 | 27.22 | 3.065 | 144 | 84.62 |
| 160 | Mewat | 568 | 15.50 | 5.16 | 5.67 | 6.17 | 6.300 | 63 | 11.12 | 6.485 | 82 | 14.37 |
| 161 | Palwal | 203 | 31.30 | 2.35 | 2.68 | 3.01 | 3.316 | 48 | 23.72 | 3.514 | 63 | 31.14 |
| 162 | Panchkula | 156 | 79.70 | 1.30 | 1.54 | 1.79 | 2.019 | 48 | 31.12 | 3.071 | 155 | 99.41 |
| 163 | Panipat | 239 | 78.10 | 1.31 | 1.56 | 1.80 | 2.034 | 73 | 30.41 | 3.068 | 231 | 96.67 |
| 164 | Rewari | 383 | 47.80 | 1.61 | 1.87 | 2.13 | 1.925 | 11 | 2.96 | 2.829 | 196 | 51.28 |
| 165 | Rohtak | 382 | 69.70 | 1.94 | 2.23 | 2.52 | 3.364 | 194 | 50.87 | 4.078 | 316 | 82.87 |
| 166 | Sirsa | 307 | 73.50 | 1.91 | 2.18 | 2.44 | 2.799 | 87 | 28.39 | 4.120 | 273 | 88.99 |
| 167 | Sonipat | 100 | 77.80 | 1.42 | 1.67 | 1.92 | 2.176 | 30 | 30.28 | 3.276 | 96 | 96.16 |
| 168 | Yamunanagar | 156 | 73.00 | 1.08 | 1.28 | 1.49 | 1.641 | 44 | 28.17 | 2.409 | 138 | 88.17 |
|  | **Himachal Pradesh** | |  |  |  |  |  |  |  |  |  |  |
| 169 | Bilaspur | 151 | 52.10 | 1.82 | 2.17 | 2.52 | 2.591 | 29 | 19.38 | 3.407 | 86 | 57.02 |
| 170 | Chamba | 177 | 51.60 | 1.67 | 1.95 | 2.24 | 2.324 | 34 | 19.18 | 3.049 | 100 | 56.34 |
| 171 | Hamirpur | 262 | 44.00 | 1.51 | 1.82 | 2.13 | 1.870 | 7 | 2.72 | 2.664 | 121 | 46.38 |
| 172 | Kangra | 254 | 47.90 | 1.34 | 1.63 | 1.92 | 1.678 | 8 | 2.97 | 2.468 | 130 | 51.41 |
| 173 | Kinnaur | 251 | 68.60 | 1.56 | 1.91 | 2.26 | 2.412 | 66 | 26.27 | 3.460 | 204 | 81.14 |
| 174 | Kullu | 288 | 63.40 | 1.64 | 1.94 | 2.24 | 2.407 | 69 | 24.06 | 3.359 | 211 | 73.16 |
| 175 | Lahul And Spiti | 218 | 62.00 | 1.42 | 1.79 | 2.17 | 2.210 | 51 | 23.47 | 3.062 | 155 | 71.07 |
| 176 | Mandi | 165 | 65.60 | 1.70 | 2.01 | 2.32 | 2.512 | 41 | 24.99 | 3.547 | 126 | 76.49 |
| 177 | Shimla | 281 | 73.30 | 1.52 | 1.84 | 2.16 | 2.361 | 79 | 28.30 | 3.471 | 249 | 88.66 |
| 178 | Sirmaur | 275 | 60.50 | 1.87 | 2.18 | 2.49 | 2.678 | 63 | 22.84 | 3.681 | 189 | 68.87 |
| 179 | Solan | 286 | 72.40 | 1.41 | 1.69 | 1.98 | 2.162 | 80 | 27.91 | 3.164 | 249 | 87.20 |
| 180 | Una | 272 | 33.90 | 1.57 | 1.86 | 2.16 | 1.899 | 6 | 2.09 | 2.495 | 93 | 34.12 |
|  | **Jammu & Kashmir** | |  |  |  |  |  |  |  |  |  |  |
| 181 | Anantnag | 149 | 54.00 | 1.49 | 1.73 | 1.98 | 2.079 | 30 | 20.15 | 2.761 | 89 | 59.62 |
| 182 | Badgam | 212 | 69.10 | 1.54 | 1.79 | 2.03 | 2.264 | 56 | 26.48 | 3.256 | 174 | 81.92 |
| 183 | Bandipora | 238 | 54.00 | 1.84 | 2.12 | 2.40 | 2.547 | 48 | 20.15 | 3.384 | 142 | 59.62 |
| 184 | Baramula | 179 | 58.60 | 1.51 | 1.78 | 2.05 | 2.172 | 39 | 22.05 | 2.957 | 118 | 66.11 |
| 185 | Doda | 247 | 30.40 | 2.52 | 2.89 | 3.26 | 3.554 | 57 | 22.96 | 3.760 | 74 | 30.12 |
| 186 | Ganderbal | 248 | 60.10 | 1.87 | 2.15 | 2.42 | 2.637 | 56 | 22.67 | 3.618 | 169 | 68.28 |
| 187 | Jammu | 151 | 63.80 | 1.41 | 1.67 | 1.93 | 2.075 | 37 | 24.22 | 2.902 | 111 | 73.76 |
| 188 | Kargil | 218 | 62.90 | 2.04 | 2.35 | 2.65 | 3.406 | 98 | 44.93 | 4.052 | 158 | 72.41 |
| 189 | Kathua | 121 | 74.10 | 1.77 | 2.04 | 2.31 | 2.624 | 35 | 28.65 | 3.875 | 109 | 89.97 |
| 190 | Kishtwar | 164 | 55.40 | 2.51 | 2.87 | 3.23 | 3.980 | 64 | 38.66 | 4.637 | 101 | 61.57 |
| 191 | Kulgam | 160 | 60.40 | 1.72 | 1.99 | 2.26 | 2.444 | 36 | 22.80 | 3.358 | 110 | 68.72 |
| 192 | Kupwara | 211 | 50.90 | 2.41 | 2.73 | 3.05 | 3.686 | 74 | 35.03 | 4.242 | 117 | 55.39 |
| 193 | Leh (Ladakh) | 248 | 70.30 | 1.85 | 2.17 | 2.50 | 2.756 | 67 | 27.00 | 3.989 | 208 | 83.82 |
| 194 | Pulwama | 325 | 69.00 | 1.31 | 1.55 | 1.79 | 1.960 | 86 | 26.44 | 2.817 | 266 | 81.76 |
| 195 | Punch | 125 | 48.60 | 2.11 | 2.38 | 2.65 | 3.312 | 49 | 39.16 | 3.625 | 65 | 52.33 |
| 196 | Rajouri | 275 | 28.00 | 2.14 | 2.44 | 2.73 | 2.952 | 58 | 20.97 | 3.110 | 75 | 27.44 |
| 197 | Ramban | 436 | 41.50 | 2.77 | 3.17 | 3.57 | 4.204 | 142 | 32.60 | 4.541 | 188 | 43.25 |
| 198 | Reasi | 273 | 53.30 | 2.06 | 2.33 | 2.61 | 3.191 | 101 | 36.95 | 3.697 | 160 | 58.66 |
| 199 | Samba | 142 | 31.20 | 1.81 | 2.10 | 2.38 | 2.140 | 3 | 1.92 | 2.751 | 44 | 31.02 |
| 200 | Shupiyan | 312 | 66.30 | 1.46 | 1.70 | 1.94 | 2.130 | 79 | 25.28 | 3.019 | 242 | 77.56 |
| 201 | Srinagar | 191 | 68.40 | 1.21 | 1.44 | 1.68 | 1.817 | 50 | 26.18 | 2.604 | 155 | 80.82 |
| 202 | Udhampur | 124 | 70.20 | 2.06 | 2.36 | 2.65 | 3.571 | 64 | 51.31 | 4.334 | 104 | 83.66 |
|  | **Jharkhand** | |  |  |  |  |  |  |  |  |  |  |
| 203 | Bokaro | 421 | 54.40 | 1.91 | 2.11 | 2.31 | 2.539 | 86 | 20.32 | 3.380 | 253 | 60.18 |
| 204 | Chatra | 390 | 36.20 | 2.96 | 3.30 | 3.64 | 4.221 | 109 | 27.91 | 4.515 | 144 | 36.82 |
| 205 | Deoghar | 347 | 46.10 | 2.62 | 2.94 | 3.26 | 4.022 | 128 | 36.82 | 4.383 | 170 | 49.07 |
| 206 | Dhanbad | 395 | 49.80 | 1.59 | 1.77 | 1.94 | 1.825 | 12 | 3.08 | 2.724 | 213 | 53.92 |
| 207 | Dumka | 248 | 36.30 | 2.25 | 2.58 | 2.91 | 3.302 | 69 | 28.00 | 3.533 | 91 | 36.94 |
| 208 | Garhwa | 121 | 34.10 | 3.04 | 3.40 | 3.77 | 4.287 | 31 | 26.10 | 4.568 | 41 | 34.35 |
| 209 | Giridih | 293 | 47.70 | 2.69 | 3.01 | 3.34 | 4.163 | 112 | 38.31 | 4.550 | 150 | 51.15 |
| 210 | Godda | 286 | 46.20 | 2.75 | 3.13 | 3.52 | 4.285 | 105 | 36.91 | 4.670 | 141 | 49.20 |
| 211 | Gumla | 218 | 26.00 | 2.13 | 2.45 | 2.76 | 2.924 | 42 | 19.34 | 3.069 | 55 | 25.25 |
| 212 | Hazaribagh | 142 | 55.80 | 2.09 | 2.37 | 2.65 | 3.294 | 55 | 38.99 | 3.842 | 88 | 62.13 |
| 213 | Jamtara | 157 | 44.00 | 2.67 | 3.00 | 3.32 | 4.046 | 55 | 34.88 | 4.391 | 73 | 46.38 |
| 214 | Khunti | 117 | 37.60 | 1.98 | 2.28 | 2.59 | 2.944 | 34 | 29.13 | 3.158 | 45 | 38.49 |
| 215 | Kodarma | 322 | 62.20 | 2.71 | 3.04 | 3.37 | 4.388 | 143 | 44.34 | 5.210 | 230 | 71.37 |
| 216 | Latehar | 243 | 26.00 | 2.44 | 2.80 | 3.16 | 3.341 | 47 | 19.34 | 3.507 | 61 | 25.25 |
| 217 | Lohardaga | 247 | 24.30 | 2.28 | 2.62 | 2.96 | 3.091 | 44 | 17.97 | 3.234 | 58 | 23.42 |
| 218 | Pakur | 122 | 35.00 | 2.76 | 3.12 | 3.47 | 3.958 | 33 | 26.87 | 4.225 | 43 | 35.40 |
| 219 | Palamu | 234 | 26.10 | 2.69 | 3.04 | 3.40 | 3.630 | 45 | 19.42 | 3.811 | 59 | 25.36 |
| 220 | Pashchimi Singhbhum | 86 | 15.90 | 2.42 | 2.76 | 3.11 | 3.075 | 10 | 11.42 | 3.167 | 13 | 14.76 |
| 221 | Purbi Singhbhum | 191 | 32.90 | 1.47 | 1.65 | 1.83 | 1.683 | 4 | 2.03 | 2.194 | 63 | 32.96 |
| 222 | Ramgarh | 338 | 45.20 | 1.86 | 2.06 | 2.25 | 2.118 | 9 | 2.80 | 3.047 | 162 | 47.91 |
| 223 | Ranchi | 140 | 44.30 | 1.71 | 1.91 | 2.12 | 1.962 | 4 | 2.74 | 2.803 | 66 | 46.76 |
| 224 | Sahibganj | 162 | 34.60 | 3.16 | 3.55 | 3.95 | 4.492 | 43 | 26.53 | 4.790 | 57 | 34.94 |
| 225 | Saraikela Kharsawan | 149 | 28.40 | 2.30 | 2.64 | 2.97 | 3.202 | 32 | 21.30 | 3.376 | 42 | 27.88 |
| 226 | Simdega | 367 | 25.70 | 2.48 | 2.86 | 3.23 | 3.406 | 70 | 19.10 | 3.573 | 91 | 24.93 |
|  | **Karnataka** | |  |  |  |  |  |  |  |  |  |  |
| 227 | Bagalkot | 203 | 54.90 | 1.74 | 2.02 | 2.30 | 2.435 | 42 | 20.52 | 3.250 | 124 | 60.87 |
| 228 | Banglore | 135 | 45.30 | 1.27 | 1.53 | 1.79 | 1.573 | 4 | 2.80 | 2.265 | 65 | 48.04 |
| 229 | Banglore Rural | 152 | 58.20 | 1.42 | 1.69 | 1.96 | 2.060 | 33 | 21.88 | 2.798 | 100 | 65.53 |
| 230 | Belgaum | 188 | 60.00 | 1.76 | 2.06 | 2.35 | 2.526 | 43 | 22.63 | 3.464 | 128 | 68.14 |
| 231 | Bellary | 225 | 50.80 | 2.00 | 2.29 | 2.59 | 3.090 | 79 | 34.95 | 3.555 | 124 | 55.26 |
| 232 | Bidar | 192 | 59.90 | 1.69 | 1.97 | 2.25 | 2.415 | 43 | 22.59 | 3.309 | 130 | 67.99 |
| 233 | Bijapur | 350 | 58.70 | 1.64 | 1.92 | 2.20 | 2.344 | 77 | 22.09 | 3.192 | 232 | 66.25 |
| 234 | Chamarajanagar | 186 | 51.70 | 1.13 | 1.38 | 1.63 | 1.645 | 36 | 19.22 | 2.159 | 105 | 56.47 |
| 235 | Chikkaballapura | 128 | 64.70 | 1.40 | 1.70 | 2.00 | 2.118 | 31 | 24.61 | 2.977 | 96 | 75.12 |
| 236 | Chikmagalur | 107 | 45.90 | 1.35 | 1.67 | 1.98 | 1.717 | 3 | 2.84 | 2.485 | 52 | 48.81 |
| 237 | Chitradurga | 127 | 64.10 | 1.14 | 1.38 | 1.62 | 1.716 | 31 | 24.35 | 2.404 | 94 | 74.21 |
| 238 | Dakshina Kannada | 144 | 21.00 | 1.56 | 1.86 | 2.17 | 1.884 | 2 | 1.29 | 2.231 | 29 | 19.94 |
| 239 | Davanagere | 196 | 57.70 | 1.83 | 2.13 | 2.43 | 2.592 | 42 | 21.67 | 3.511 | 127 | 64.82 |
| 240 | Dharwad | 169 | 59.30 | 1.68 | 1.98 | 2.28 | 2.422 | 38 | 22.34 | 3.309 | 113 | 67.12 |
| 241 | Gadag | 173 | 59.60 | 1.18 | 1.42 | 1.66 | 1.739 | 39 | 22.46 | 2.379 | 117 | 67.55 |
| 242 | Gulbarga | 291 | 53.70 | 1.84 | 2.12 | 2.41 | 2.545 | 58 | 20.03 | 3.375 | 172 | 59.21 |
| 243 | Hassan | 606 | 56.70 | 1.49 | 1.80 | 2.11 | 2.183 | 129 | 21.26 | 2.941 | 384 | 63.40 |
| 244 | Haveri | 135 | 48.60 | 1.35 | 1.61 | 1.88 | 1.658 | 4 | 3.01 | 2.453 | 70 | 52.33 |
| 245 | Kodagu | 333 | 41.90 | 1.66 | 1.97 | 2.30 | 2.021 | 9 | 2.59 | 2.832 | 146 | 43.74 |
| 246 | Kolar | 435 | 62.70 | 1.55 | 1.83 | 2.12 | 2.265 | 103 | 23.76 | 3.150 | 314 | 72.11 |
| 247 | Koppal | 243 | 44.50 | 1.72 | 2.01 | 2.30 | 2.065 | 7 | 2.75 | 2.955 | 114 | 47.02 |
| 248 | Mandya | 124 | 59.40 | 1.41 | 1.71 | 2.01 | 2.093 | 28 | 22.38 | 2.860 | 83 | 67.26 |
| 249 | Mysore | 305 | 55.60 | 1.57 | 1.86 | 2.16 | 2.247 | 63 | 20.81 | 3.010 | 189 | 61.85 |
| 250 | Raichur | 195 | 54.30 | 1.80 | 2.08 | 2.35 | 2.502 | 40 | 20.28 | 3.329 | 117 | 60.04 |
| 251 | Ramanagara | 153 | 55.90 | 1.24 | 1.51 | 1.79 | 1.826 | 32 | 20.93 | 2.450 | 95 | 62.27 |
| 252 | Shimoga | 429 | 36.80 | 1.42 | 1.71 | 2.00 | 1.749 | 10 | 2.27 | 2.352 | 161 | 37.53 |
| 253 | Tumkur | 267 | 62.70 | 1.20 | 1.46 | 1.72 | 1.807 | 64 | 23.76 | 2.513 | 193 | 72.11 |
| 254 | Udupi | 562 | 32.40 | 1.28 | 1.55 | 1.82 | 1.581 | 11 | 2.00 | 2.052 | 182 | 32.39 |
| 255 | Uttara Kannada | 211 | 31.30 | 1.39 | 1.68 | 1.97 | 1.712 | 4 | 1.93 | 2.203 | 66 | 31.14 |
| 256 | Yadgir | 239 | 47.00 | 2.00 | 2.30 | 2.60 | 3.166 | 90 | 37.66 | 3.455 | 120 | 50.23 |
|  | **Kerala** |  |  |  |  |  |  |  |  |  |  |  |
| 257 | Alappuzha | 82 | 45.10 | 1.11 | 1.42 | 1.72 | 1.460 | 2 | 2.79 | 2.099 | 39 | 47.78 |
| 258 | Ernakulam | 108 | 57.80 | 1.27 | 1.58 | 1.88 | 1.923 | 23 | 21.72 | 2.606 | 70 | 64.96 |
| 259 | Idukki | 168 | 63.00 | 0.97 | 1.25 | 1.53 | 1.549 | 40 | 23.89 | 2.157 | 122 | 72.56 |
| 260 | Kannur | 160 | 49.20 | 1.36 | 1.67 | 1.99 | 1.721 | 5 | 3.05 | 2.557 | 85 | 53.12 |
| 261 | Kasaragod | 175 | 42.60 | 1.32 | 1.61 | 1.90 | 1.652 | 5 | 2.63 | 2.328 | 78 | 44.62 |
| 262 | Kollam | 187 | 53.10 | 1.17 | 1.46 | 1.75 | 1.749 | 37 | 19.79 | 2.312 | 109 | 58.38 |
| 263 | Kottayam | 113 | 52.90 | 1.21 | 1.51 | 1.81 | 1.808 | 22 | 19.70 | 2.387 | 66 | 58.11 |
| 264 | Kozhikode | 116 | 57.50 | 1.18 | 1.46 | 1.73 | 1.775 | 25 | 21.59 | 2.402 | 75 | 64.53 |
| 265 | Mallapuram | 298 | 43.10 | 1.43 | 1.70 | 1.97 | 1.745 | 8 | 2.66 | 2.469 | 135 | 45.24 |
| 266 | Palakkad | 284 | 62.20 | 1.32 | 1.61 | 1.89 | 1.989 | 67 | 23.55 | 2.759 | 203 | 71.37 |
| 267 | Pathanamthitta | 647 | 50.90 | 1.26 | 1.60 | 1.94 | 1.902 | 122 | 18.89 | 2.486 | 358 | 55.39 |
| 268 | Thiruvanthapuram | 162 | 49.10 | 1.34 | 1.65 | 1.97 | 1.700 | 5 | 3.04 | 2.524 | 86 | 52.99 |
| 269 | Thrissur | 91 | 63.80 | 1.15 | 1.44 | 1.74 | 1.789 | 22 | 24.22 | 2.502 | 67 | 73.76 |
| 270 | Wayanad | 86 | 57.80 | 1.33 | 1.61 | 1.89 | 1.960 | 19 | 21.72 | 2.656 | 56 | 64.96 |
|  | **Lakshadweep** | |  |  |  |  |  |  |  |  |  |  |
| 271 | Lakshadweep | 213 | 29.70 | 1.55 | 1.82 | 2.07 | 1.853 | 4 | 1.83 | 2.354 | 63 | 29.33 |
|  | **Madhya Pradesh** | |  |  |  |  |  |  |  |  |  |  |
| 272 | Alirajpur | 477 | 30.90 | 3.20 | 3.52 | 3.84 | 4.343 | 112 | 23.38 | 4.600 | 146 | 30.68 |
| 273 | Anuppur | 243 | 49.10 | 1.98 | 2.27 | 2.56 | 3.170 | 96 | 39.64 | 3.473 | 129 | 52.99 |
| 274 | Ashoknagar | 270 | 58.30 | 2.25 | 2.56 | 2.87 | 3.611 | 111 | 41.05 | 4.241 | 177 | 65.68 |
| 275 | Balaghat | 227 | 58.20 | 1.86 | 2.15 | 2.43 | 2.620 | 50 | 21.88 | 3.559 | 149 | 65.53 |
| 276 | Barwani | 423 | 50.70 | 2.78 | 3.08 | 3.38 | 4.154 | 148 | 34.87 | 4.778 | 233 | 55.13 |
| 277 | Betul | 201 | 64.00 | 1.56 | 1.81 | 2.06 | 2.250 | 49 | 24.31 | 3.151 | 149 | 74.06 |
| 278 | Bhind | 283 | 55.70 | 2.31 | 2.62 | 2.92 | 3.639 | 110 | 38.91 | 4.244 | 175 | 61.99 |
| 279 | Bhopal | 210 | 52.90 | 1.60 | 1.85 | 2.11 | 2.215 | 41 | 19.70 | 2.925 | 122 | 58.11 |
| 280 | Burhanpur | 608 | 56.40 | 2.39 | 2.60 | 2.81 | 3.627 | 240 | 39.48 | 4.237 | 383 | 62.97 |
| 281 | Chhatarpur | 273 | 53.90 | 2.42 | 2.76 | 3.09 | 3.793 | 102 | 37.44 | 4.402 | 162 | 59.48 |
| 282 | Chhindwara | 224 | 66.10 | 1.67 | 1.93 | 2.19 | 2.416 | 57 | 25.20 | 3.421 | 173 | 77.26 |
| 283 | Damoh | 223 | 36.20 | 1.87 | 2.15 | 2.43 | 2.198 | 5 | 2.23 | 2.942 | 82 | 36.82 |
| 284 | Datia | 272 | 59.00 | 1.15 | 2.44 | 2.73 | 3.456 | 113 | 41.64 | 4.067 | 181 | 66.69 |
| 285 | Dewas | 275 | 56.60 | 2.16 | 2.45 | 2.74 | 3.421 | 109 | 39.65 | 4.000 | 174 | 63.26 |
| 286 | Dhar | 292 | 52.60 | 1.97 | 2.23 | 2.48 | 3.041 | 106 | 36.39 | 3.517 | 169 | 57.70 |
| 287 | Dindori | 229 | 66.80 | 1.93 | 2.22 | 2.51 | 3.292 | 110 | 48.31 | 3.959 | 179 | 78.33 |
| 288 | Guna | 241 | 60.90 | 2.22 | 2.50 | 2.77 | 3.581 | 104 | 43.23 | 4.236 | 167 | 69.45 |
| 289 | Gwalior | 198 | 49.20 | 2.01 | 2.20 | 2.40 | 2.267 | 6 | 3.05 | 3.369 | 105 | 53.12 |
| 290 | Harda | 150 | 49.40 | 1.93 | 2.20 | 2.46 | 2.267 | 5 | 3.06 | 3.375 | 80 | 53.39 |
| 291 | Hoshangabad | 228 | 50.20 | 1.90 | 2.08 | 2.26 | 2.467 | 42 | 18.61 | 3.213 | 124 | 54.45 |
| 292 | Indore | 442 | 54.00 | 1.73 | 1.90 | 2.07 | 2.283 | 89 | 20.15 | 3.033 | 263 | 59.62 |
| 293 | Jabalpur | 490 | 65.90 | 1.67 | 1.85 | 2.04 | 2.315 | 123 | 25.11 | 3.274 | 377 | 76.95 |
| 294 | Jhabua | 321 | 10.40 | 3.14 | 3.52 | 3.89 | 3.778 | 24 | 7.33 | 3.852 | 30 | 9.42 |
| 295 | Katni | 359 | 45.70 | 1.85 | 2.14 | 2.43 | 2.200 | 10 | 2.83 | 3.179 | 174 | 48.55 |
| 296 | Khandwa (East Nimar) | 135 | 67.30 | 2.19 | 2.47 | 2.75 | 3.674 | 66 | 48.75 | 4.424 | 107 | 79.11 |
| 297 | Khargone (West Nimar) | 198 | 70.60 | 1.96 | 2.23 | 2.50 | 3.382 | 103 | 51.67 | 4.110 | 167 | 84.30 |
| 298 | Mandla | 196 | 66.90 | 1.68 | 1.95 | 2.23 | 2.448 | 50 | 25.54 | 3.481 | 154 | 78.49 |
| 299 | Mandsaur | 260 | 18.10 | 1.85 | 2.11 | 2.37 | 2.133 | 3 | 1.11 | 2.468 | 44 | 16.97 |
| 300 | Morena | 289 | 56.30 | 2.34 | 2.64 | 2.95 | 3.680 | 114 | 39.40 | 4.299 | 182 | 62.83 |
| 301 | Narsimhapur | 203 | 51.00 | 1.57 | 1.82 | 2.07 | 2.165 | 38 | 18.93 | 2.831 | 113 | 55.53 |
| 302 | Neemuch | 79 | 17.60 | 1.71 | 1.97 | 2.24 | 1.991 | 1 | 1.08 | 2.294 | 13 | 16.46 |
| 303 | Panna | 354 | 45.90 | 2.18 | 2.51 | 2.84 | 3.429 | 130 | 36.63 | 3.735 | 173 | 48.81 |
| 304 | Raisen | 275 | 66.00 | 2.16 | 2.45 | 2.74 | 3.616 | 131 | 47.61 | 4.339 | 212 | 77.10 |
| 305 | Rajgarh | 145 | 53.50 | 2.35 | 2.68 | 3.00 | 3.675 | 54 | 37.12 | 4.259 | 85 | 58.93 |
| 306 | Ratlam | 114 | 24.80 | 1.98 | 2.27 | 2.55 | 2.687 | 21 | 18.37 | 2.814 | 27 | 23.96 |
| 307 | Rewa | 197 | 59.30 | 2.14 | 2.44 | 2.73 | 3.462 | 83 | 41.89 | 4.078 | 133 | 67.12 |
| 308 | Sagar | 645 | 49.00 | 2.43 | 2.76 | 3.09 | 3.851 | 255 | 39.54 | 4.219 | 341 | 52.86 |
| 309 | Satna | 269 | 54.90 | 1.98 | 2.29 | 2.59 | 3.166 | 103 | 38.25 | 3.684 | 164 | 60.87 |
| 310 | Sehore | 388 | 55.10 | 2.17 | 2.47 | 2.77 | 3.419 | 149 | 38.42 | 3.980 | 237 | 61.15 |
| 311 | Seoni | 137 | 70.00 | 1.54 | 1.79 | 2.05 | 2.271 | 37 | 26.87 | 3.282 | 114 | 83.35 |
| 312 | Shahdol | 237 | 43.50 | 1.79 | 2.09 | 2.38 | 2.146 | 6 | 2.69 | 3.046 | 108 | 45.75 |
| 313 | Shajapur | 430 | 55.50 | 1.96 | 2.25 | 2.54 | 3.122 | 167 | 38.74 | 3.638 | 266 | 61.71 |
| 314 | Sheopur | 131 | 53.20 | 2.53 | 2.85 | 3.18 | 3.901 | 48 | 36.87 | 4.518 | 77 | 58.52 |
| 315 | Shivpuri | 191 | 57.70 | 1.18 | 2.47 | 2.77 | 3.472 | 77 | 40.56 | 4.071 | 124 | 64.82 |
| 316 | Sidhi | 227 | 32.50 | 2.68 | 3.02 | 3.37 | 3.767 | 56 | 24.73 | 4.002 | 74 | 32.50 |
| 317 | Singrauli | 309 | 37.10 | 2.77 | 3.09 | 3.41 | 3.977 | 89 | 28.70 | 4.261 | 117 | 37.89 |
| 318 | Tikamgarh | 234 | 48.50 | 1.80 | 2.07 | 2.33 | 2.132 | 7 | 3.00 | 3.150 | 122 | 52.20 |
| 319 | Ujjain | 261 | 35.10 | 1.95 | 2.13 | 2.31 | 2.176 | 6 | 2.16 | 2.887 | 93 | 35.52 |
| 320 | Umaria | 152 | 52.30 | 2.19 | 2.51 | 2.83 | 3.417 | 55 | 36.15 | 3.948 | 87 | 57.29 |
| 321 | Vidisha | 182 | 23.00 | 2.46 | 2.80 | 3.13 | 3.274 | 31 | 16.93 | 3.417 | 40 | 22.04 |
|  | **Maharashtra** | |  |  |  |  |  |  |  |  |  |  |
| 322 | Ahmednagar | 174 | 48.40 | 1.79 | 2.10 | 2.42 | 2.163 | 5 | 3.00 | 3.193 | 90 | 52.07 |
| 323 | Akola | 179 | 67.40 | 1.57 | 1.84 | 2.11 | 2.314 | 46 | 25.75 | 3.298 | 142 | 79.26 |
| 324 | Amravati | 149 | 72.40 | 1.37 | 1.64 | 1.90 | 2.098 | 42 | 27.91 | 3.070 | 130 | 87.20 |
| 325 | Aurangabad | 302 | 64.60 | 1.84 | 2.15 | 2.45 | 2.678 | 74 | 24.56 | 3.762 | 227 | 74.97 |
| 326 | Bhandara | 116 | 77.40 | 1.28 | 1.57 | 1.86 | 2.043 | 35 | 30.10 | 3.069 | 111 | 95.48 |
| 327 | Bid | 206 | 67.10 | 1.99 | 2.31 | 2.63 | 3.432 | 100 | 48.57 | 4.130 | 163 | 78.80 |
| 328 | Buldhana | 147 | 73.40 | 1.51 | 1.80 | 2.09 | 2.310 | 42 | 28.35 | 3.399 | 131 | 88.82 |
| 329 | Chandrapur | 152 | 73.20 | 1.55 | 1.84 | 2.14 | 2.360 | 43 | 28.26 | 3.468 | 135 | 88.50 |
| 330 | Dhule | 182 | 63.80 | 1.85 | 2.17 | 2.48 | 2.696 | 44 | 24.22 | 3.771 | 134 | 73.76 |
| 331 | Gadchiroli | 138 | 75.10 | 1.23 | 1.50 | 1.77 | 1.936 | 40 | 29.09 | 2.874 | 126 | 91.62 |
| 332 | Gondiya | 375 | 67.70 | 1.63 | 1.94 | 2.24 | 2.442 | 97 | 25.88 | 3.487 | 299 | 79.73 |
| 333 | Hingoli | 284 | 66.90 | 1.86 | 2.17 | 2.47 | 2.724 | 73 | 25.54 | 3.873 | 223 | 78.49 |
| 334 | Jalgaon | 177 | 65.30 | 1.92 | 2.26 | 2.60 | 3.322 | 83 | 47.00 | 3.978 | 134 | 76.03 |
| 335 | Jalna | 174 | 65.00 | 2.12 | 2.45 | 2.77 | 3.595 | 81 | 46.74 | 4.302 | 132 | 75.58 |
| 336 | Kolhapur | 106 | 53.70 | 1.19 | 1.46 | 1.73 | 1.752 | 21 | 20.03 | 2.324 | 63 | 59.21 |
| 337 | Latur | 214 | 67.40 | 2.09 | 2.39 | 2.69 | 3.557 | 105 | 48.83 | 4.284 | 170 | 79.26 |
| 338 | Mumbai | 104 | 59.60 | 1.41 | 1.76 | 2.12 | 2.155 | 23 | 22.46 | 2.949 | 70 | 67.55 |
| 339 | Mumbai Suburban | 314 | 64.90 | 1.22 | 1.51 | 1.80 | 1.883 | 78 | 24.69 | 2.649 | 237 | 75.42 |
| 340 | Nagpur | 408 | 69.10 | 1.34 | 1.58 | 1.81 | 1.998 | 108 | 26.48 | 2.874 | 335 | 81.92 |
| 341 | Nanded | 207 | 60.90 | 1.91 | 2.19 | 2.47 | 2.694 | 48 | 23.01 | 3.711 | 144 | 69.45 |
| 342 | Nandurbar | 374 | 55.30 | 1.85 | 2.15 | 2.44 | 2.595 | 77 | 20.69 | 3.471 | 229 | 61.43 |
| 343 | Nashik | 132 | 67.50 | 1.75 | 2.04 | 2.32 | 2.566 | 34 | 25.80 | 3.660 | 105 | 79.42 |
| 344 | Osmanabad | 315 | 70.30 | 1.78 | 2.11 | 2.43 | 2.680 | 85 | 27.00 | 3.879 | 264 | 83.82 |
| 345 | Parbhani | 375 | 69.60 | 1.81 | 2.09 | 2.38 | 2.648 | 100 | 26.70 | 3.819 | 310 | 82.71 |
| 346 | Pune | 437 | 70.60 | 1.37 | 1.67 | 1.96 | 2.123 | 118 | 27.13 | 3.078 | 368 | 84.30 |
| 347 | Raigarh | 473 | 63.60 | 1.64 | 1.96 | 2.27 | 2.433 | 114 | 24.14 | 3.400 | 348 | 73.46 |
| 348 | Ratnagiri | 228 | 38.30 | 1.58 | 1.93 | 2.29 | 1.976 | 5 | 2.36 | 2.689 | 90 | 39.33 |
| 349 | Sangli | 367 | 52.00 | 1.47 | 1.76 | 2.05 | 2.100 | 71 | 19.34 | 2.761 | 209 | 56.88 |
| 350 | Satara | 301 | 62.50 | 1.80 | 2.15 | 2.50 | 2.659 | 71 | 23.68 | 3.694 | 216 | 71.82 |
| 351 | Sindhudurg | 193 | 50.00 | 1.18 | 1.51 | 1.84 | 1.557 | 6 | 3.10 | 2.328 | 105 | 54.19 |
| 352 | Solapur | 174 | 64.40 | 1.57 | 1.84 | 2.11 | 2.290 | 42 | 24.48 | 3.214 | 130 | 74.67 |
| 353 | Thane | 151 | 65.50 | 1.50 | 1.80 | 2.10 | 2.249 | 38 | 24.94 | 3.174 | 116 | 76.34 |
| 354 | Wardha | 173 | 78.20 | 1.27 | 1.55 | 1.83 | 2.022 | 53 | 30.46 | 3.051 | 168 | 96.84 |
| 355 | Washim | 129 | 75.50 | 1.68 | 1.98 | 2.28 | 2.559 | 38 | 29.27 | 3.807 | 119 | 92.29 |
| 356 | Yawatmal | 179 | 71.60 | 1.68 | 1.97 | 2.26 | 2.513 | 49 | 27.56 | 3.662 | 154 | 85.90 |
|  | **Manipur** |  |  |  |  |  |  |  |  |  |  |  |
| 357 | Bishnupur | 515 | 32.70 | 2.20 | 2.41 | 2.62 | 3.010 | 128 | 24.90 | 3.199 | 169 | 32.73 |
| 358 | Chandel | 276 | 15.40 | 2.68 | 3.04 | 3.40 | 3.376 | 31 | 11.04 | 3.474 | 39 | 14.27 |
| 359 | Churachandpur | 246 | 23.60 | 2.68 | 3.07 | 3.46 | 3.604 | 43 | 17.41 | 3.766 | 56 | 22.68 |
| 360 | Imphal East | 76 | 30.90 | 1.98 | 2.18 | 2.38 | 2.221 | 1 | 1.90 | 2.849 | 23 | 30.68 |
| 361 | Imphal West | 462 | 20.40 | 2.06 | 2.28 | 2.49 | 2.619 | 69 | 14.88 | 2.721 | 89 | 19.32 |
| 362 | Senapati | 196 | 17.50 | 2.99 | 3.36 | 3.72 | 3.785 | 25 | 12.64 | 3.910 | 32 | 16.36 |
| 363 | Tamenglong | 146 | 16.20 | 3.40 | 3.81 | 4.21 | 4.254 | 17 | 11.65 | 4.384 | 22 | 15.06 |
| 364 | Thoubal | 549 | 24.00 | 2.38 | 2.60 | 2.81 | 3.061 | 97 | 17.73 | 3.201 | 127 | 23.10 |
| 365 | Ukhrul | 242 | 12.70 | 3.01 | 3.43 | 3.85 | 3.739 | 22 | 9.02 | 3.829 | 28 | 11.63 |
|  | **Meghalaya** | |  |  |  |  |  |  |  |  |  |  |
| 366 | East Garo Hills | 320 | 12.80 | 2.60 | 2.93 | 3.26 | 3.196 | 29 | 9.09 | 3.273 | 37 | 11.72 |
| 367 | East Khasi Hills | 594 | 24.90 | 2.73 | 2.98 | 3.22 | 3.530 | 110 | 18.45 | 3.697 | 143 | 24.06 |
| 368 | Jaintia Hills | 156 | 20.90 | 4.06 | 4.48 | 4.89 | 5.164 | 24 | 15.27 | 5.369 | 31 | 19.84 |
| 369 | Ribhoi | 237 | 23.70 | 3.30 | 3.69 | 4.07 | 4.335 | 41 | 17.49 | 4.531 | 54 | 22.78 |
| 370 | South Garo Hills | 201 | 21.80 | 1.44 | 1.69 | 1.93 | 1.713 | 3 | 1.34 | 2.041 | 42 | 20.78 |
| 371 | West Garo Hills | 112 | 34.00 | 1.62 | 1.88 | 2.14 | 1.919 | 2 | 2.10 | 2.524 | 38 | 34.24 |
| 372 | West Khasi Hills | 277 | 21.90 | 3.98 | 4.43 | 4.86 | 5.141 | 44 | 16.06 | 5.355 | 58 | 20.88 |
|  | **Mizoram** |  |  |  |  |  |  |  |  |  |  |  |
| 373 | Aizawl | 200 | 40.90 | 1.76 | 2.05 | 2.33 | 2.102 | 5 | 2.53 | 2.921 | 85 | 42.50 |
| 374 | Champhai | 117 | 32.40 | 2.71 | 2.97 | 3.22 | 3.702 | 29 | 24.65 | 3.932 | 38 | 32.39 |
| 375 | Kolasib | 112 | 33.50 | 2.44 | 2.70 | 2.96 | 3.391 | 29 | 25.58 | 3.609 | 38 | 33.66 |
| 376 | Lawngtlai | 176 | 27.10 | 1.95 | 2.27 | 2.57 | 2.729 | 36 | 20.24 | 2.870 | 47 | 26.45 |
| 377 | Lunglei | 450 | 29.00 | 1.76 | 1.97 | 2.18 | 2.005 | 8 | 1.78 | 2.532 | 129 | 28.55 |
| 378 | Mamit | 229 | 34.30 | 2.32 | 2.67 | 3.02 | 3.371 | 60 | 26.27 | 3.593 | 79 | 34.59 |
| 379 | Saiha | 391 | 33.40 | 2.53 | 2.77 | 3.00 | 3.476 | 100 | 25.50 | 3.699 | 131 | 33.54 |
| 380 | Serchhip | 341 | 41.20 | 2.04 | 2.27 | 2.49 | 3.004 | 110 | 32.33 | 3.243 | 146 | 42.87 |
|  | **Nagaland** |  |  |  |  |  |  |  |  |  |  |  |
| 381 | Dimapur | 437 | 27.40 | 1.93 | 2.14 | 2.34 | 2.176 | 7 | 1.69 | 2.713 | 117 | 26.78 |
| 382 | Kiphire | 420 | 31.10 | 2.98 | 3.41 | 3.84 | 4.213 | 99 | 23.55 | 4.464 | 130 | 30.91 |
| 383 | Kohima | 214 | 33.80 | 1.70 | 1.92 | 2.13 | 1.960 | 4 | 2.08 | 2.573 | 73 | 34.01 |
| 384 | Longleng | 168 | 20.90 | 2.24 | 2.61 | 2.99 | 3.009 | 26 | 15.27 | 3.128 | 33 | 19.84 |
| 385 | Mokokchung | 107 | 38.20 | 1.55 | 1.92 | 2.28 | 1.965 | 3 | 2.36 | 2.673 | 42 | 39.21 |
| 386 | Mon | 338 | 12.50 | 3.41 | 3.83 | 4.25 | 4.170 | 30 | 8.87 | 4.268 | 39 | 11.43 |
| 387 | Paren | 154 | 31.00 | 2.86 | 3.26 | 3.67 | 4.025 | 36 | 23.47 | 4.264 | 47 | 30.79 |
| 388 | Phek | 302 | 26.20 | 2.79 | 3.19 | 3.58 | 3.812 | 59 | 19.50 | 4.002 | 77 | 25.47 |
| 389 | Tuensang | 120 | 21.20 | 3.43 | 3.92 | 4.40 | 4.528 | 19 | 15.51 | 4.710 | 24 | 20.15 |
| 390 | Wokha | 145 | 30.70 | 2.12 | 2.54 | 2.95 | 3.130 | 34 | 23.21 | 3.314 | 44 | 30.46 |
| 391 | Zunheboto | 183 | 32.50 | 2.31 | 2.70 | 3.09 | 3.368 | 45 | 24.73 | 3.578 | 60 | 32.50 |
|  | **NCT Of Delhi** | |  |  |  |  |  |  |  |  |  |  |
| 392 | Central Delhi | 65 | 48.00 | 0.76 | 1.01 | 1.26 | 1.040 | 2 | 2.97 | 1.531 | 33 | 51.54 |
| 393 | East Delhi | 113 | 38.10 | 1.44 | 1.77 | 2.10 | 1.812 | 3 | 2.35 | 2.462 | 44 | 39.09 |
| 394 | New Delhi | 155 | 42.30 | 1.25 | 1.62 | 1.99 | 1.662 | 4 | 2.61 | 2.337 | 69 | 44.24 |
| 395 | North Delhi | 113 | 46.00 | 1.62 | 1.95 | 2.29 | 2.005 | 3 | 2.85 | 2.904 | 55 | 48.94 |
| 396 | North East Delhi | 171 | 54.00 | 1.69 | 2.00 | 2.30 | 2.403 | 34 | 20.15 | 3.192 | 102 | 59.62 |
| 397 | North West Delhi | 258 | 53.80 | 1.27 | 1.59 | 1.91 | 1.909 | 52 | 20.07 | 2.534 | 153 | 59.35 |
| 398 | South Delhi | 124 | 48.10 | 1.32 | 1.65 | 1.97 | 1.699 | 4 | 2.98 | 2.503 | 64 | 51.67 |
| 399 | South West Delhi | 156 | 65.60 | 1.92 | 2.29 | 2.65 | 3.372 | 74 | 47.26 | 4.042 | 119 | 76.49 |
| 400 | West Delhi | 116 | 61.70 | 1.02 | 1.30 | 1.58 | 1.603 | 27 | 23.34 | 2.218 | 82 | 70.63 |
|  | **Odisha** |  |  |  |  |  |  |  |  |  |  |  |
| 401 | Anugul | 215 | 67.00 | 1.72 | 1.98 | 2.25 | 2.487 | 55 | 25.58 | 3.537 | 169 | 78.64 |
| 402 | Balangir | 224 | 65.30 | 2.04 | 2.34 | 2.65 | 3.440 | 105 | 47.00 | 4.119 | 170 | 76.03 |
| 403 | Baleshwar | 178 | 38.50 | 1.55 | 1.81 | 2.08 | 1.853 | 4 | 2.38 | 2.526 | 71 | 39.57 |
| 404 | Bargarh | 196 | 67.10 | 1.54 | 1.80 | 2.06 | 2.261 | 50 | 25.63 | 3.218 | 154 | 78.80 |
| 405 | Baudh | 219 | 64.50 | 1.93 | 2.23 | 2.53 | 3.263 | 102 | 46.31 | 3.898 | 164 | 74.82 |
| 406 | Bhadrak | 187 | 29.30 | 1.57 | 1.83 | 2.09 | 1.863 | 3 | 1.80 | 2.359 | 54 | 28.88 |
| 407 | Cuttack | 149 | 49.80 | 1.45 | 1.73 | 2.01 | 1.783 | 5 | 3.08 | 2.663 | 80 | 53.92 |
| 408 | Debagarh | 215 | 44.70 | 2.05 | 2.36 | 2.68 | 3.198 | 77 | 35.52 | 3.476 | 102 | 47.27 |
| 409 | Dhenkanal | 180 | 69.20 | 1.56 | 1.83 | 2.10 | 2.315 | 48 | 26.53 | 3.332 | 148 | 82.08 |
| 410 | Gajapati | 221 | 65.80 | 2.04 | 2.35 | 2.67 | 3.465 | 105 | 47.44 | 4.155 | 170 | 76.80 |
| 411 | Ganjam | 172 | 59.10 | 1.64 | 1.92 | 2.21 | 2.347 | 38 | 22.26 | 3.203 | 115 | 66.83 |
| 412 | Jagatsinghpur | 389 | 63.80 | 1.33 | 1.57 | 1.82 | 1.950 | 94 | 24.22 | 2.728 | 287 | 73.76 |
| 413 | Jajapur | 344 | 58.90 | 1.72 | 2.00 | 2.28 | 2.443 | 76 | 22.17 | 3.331 | 229 | 66.54 |
| 414 | Jharsuguda | 386 | 74.00 | 1.39 | 1.56 | 1.73 | 2.006 | 110 | 28.61 | 2.961 | 346 | 89.81 |
| 415 | Kalahandi | 232 | 61.60 | 2.14 | 2.48 | 2.83 | 3.567 | 102 | 43.83 | 4.228 | 164 | 70.48 |
| 416 | Kandhamal | 132 | 60.40 | 2.13 | 2.44 | 2.75 | 3.485 | 57 | 42.81 | 4.117 | 91 | 68.72 |
| 417 | Kendrapara | 286 | 62.80 | 1.73 | 2.00 | 2.28 | 2.476 | 68 | 23.80 | 3.445 | 207 | 72.26 |
| 418 | Kendujhar | 209 | 39.80 | 2.05 | 2.35 | 2.67 | 3.080 | 65 | 31.08 | 3.317 | 86 | 41.15 |
| 419 | Khordha | 226 | 64.40 | 1.59 | 1.79 | 1.99 | 2.228 | 55 | 24.48 | 3.127 | 168 | 74.67 |
| 420 | Koraput | 519 | 59.20 | 2.24 | 2.56 | 2.90 | 3.630 | 217 | 41.80 | 4.275 | 347 | 66.97 |
| 421 | Malkangiri | 241 | 52.00 | 2.51 | 2.83 | 3.16 | 3.846 | 86 | 35.91 | 4.440 | 137 | 56.88 |
| 422 | Mayurbhanj | 313 | 41.20 | 1.97 | 2.27 | 2.59 | 3.004 | 101 | 32.33 | 3.243 | 134 | 42.87 |
| 423 | Nabarangapur | 162 | 64.40 | 2.38 | 2.68 | 2.99 | 3.919 | 75 | 46.22 | 4.681 | 121 | 74.67 |
| 424 | Nayagarh | 228 | 62.90 | 1.61 | 1.89 | 2.17 | 2.341 | 54 | 23.84 | 3.259 | 165 | 72.41 |
| 425 | Nuapada | 162 | 58.80 | 2.26 | 2.58 | 2.90 | 3.650 | 67 | 41.47 | 4.293 | 108 | 66.40 |
| 426 | Puri | 439 | 70.50 | 1.55 | 1.81 | 2.07 | 2.300 | 119 | 27.09 | 3.333 | 369 | 84.14 |
| 427 | Rayagada | 297 | 54.90 | 2.07 | 2.38 | 2.70 | 3.290 | 114 | 38.25 | 3.829 | 181 | 60.87 |
| 428 | Sambalpur | 175 | 60.30 | 1.57 | 1.85 | 2.13 | 2.271 | 40 | 22.75 | 3.119 | 120 | 68.57 |
| 429 | Subarnapur | 269 | 60.70 | 1.47 | 1.71 | 1.96 | 2.102 | 62 | 22.92 | 2.893 | 186 | 69.16 |
| 430 | Sundargarh | 483 | 63.30 | 1.68 | 1.86 | 2.06 | 2.307 | 116 | 24.01 | 3.218 | 353 | 73.01 |
|  | **Puducherry** | |  |  |  |  |  |  |  |  |  |  |
| 431 | Karaikal | 108 | 48.70 | 1.65 | 1.94 | 2.24 | 1.998 | 3 | 3.02 | 2.958 | 57 | 52.46 |
| 432 | Mahe | 186 | 40.90 | 1.36 | 1.62 | 1.89 | 1.661 | 5 | 2.53 | 2.309 | 79 | 42.50 |
| 433 | Puduchherry | 175 | 65.00 | 1.38 | 1.64 | 1.89 | 2.046 | 43 | 24.73 | 2.879 | 132 | 75.58 |
| 434 | Yanam | 166 | 70.60 | 1.36 | 1.61 | 1.86 | 2.047 | 45 | 27.13 | 2.967 | 140 | 84.30 |
|  | **Punjab** |  |  |  |  |  |  |  |  |  |  |  |
| 435 | Amritsar | 167 | 81.00 | 1.38 | 1.62 | 1.87 | 2.134 | 53 | 31.71 | 3.267 | 170 | 101.67 |
| 436 | Barnala | 144 | 78.60 | 1.28 | 1.53 | 1.79 | 1.999 | 44 | 30.64 | 3.022 | 140 | 97.52 |
| 437 | Bathinda | 159 | 82.60 | 1.37 | 1.61 | 1.87 | 2.132 | 51 | 32.42 | 3.292 | 166 | 104.48 |
| 438 | Faridkot | 210 | 81.50 | 1.13 | 1.35 | 1.58 | 1.781 | 67 | 31.93 | 2.734 | 216 | 102.54 |
| 439 | Fatehgarh Sahib | 238 | 73.80 | 1.31 | 1.58 | 1.86 | 2.031 | 68 | 28.52 | 2.994 | 213 | 89.48 |
| 440 | Firozpur | 643 | 80.90 | 1.46 | 1.72 | 1.98 | 2.265 | 204 | 31.66 | 3.466 | 652 | 101.49 |
| 441 | Gurdaspur | 133 | 74.50 | 1.45 | 1.71 | 1.98 | 2.203 | 38 | 28.83 | 3.260 | 120 | 90.63 |
| 442 | Hoshiarpur | 522 | 70.30 | 1.41 | 1.68 | 1.95 | 2.134 | 141 | 27.00 | 3.088 | 438 | 83.82 |
| 443 | Jalandhar | 197 | 70.60 | 1.18 | 1.42 | 1.66 | 1.805 | 53 | 27.13 | 2.617 | 166 | 84.30 |
| 444 | Kapurthala | 372 | 70.00 | 1.06 | 1.30 | 1.55 | 1.649 | 100 | 26.87 | 2.383 | 310 | 83.35 |
| 445 | Ludhiana | 344 | 72.80 | 1.30 | 1.55 | 1.80 | 1.985 | 97 | 28.08 | 2.912 | 302 | 87.85 |
| 446 | Mansa | 176 | 78.30 | 1.47 | 1.72 | 1.99 | 2.245 | 54 | 30.50 | 3.389 | 171 | 97.01 |
| 447 | Moga | 182 | 76.60 | 1.58 | 1.85 | 2.12 | 2.400 | 54 | 29.75 | 3.591 | 172 | 94.13 |
| 448 | Muktsar | 97 | 84.80 | 1.24 | 1.47 | 1.71 | 1.961 | 33 | 33.42 | 3.064 | 106 | 108.42 |
| 449 | Patiala | 134 | 79.10 | 1.49 | 1.73 | 1.99 | 2.264 | 41 | 30.86 | 3.432 | 132 | 98.38 |
| 450 | Rupnagar | 180 | 75.00 | 1.43 | 1.70 | 1.98 | 2.194 | 52 | 29.05 | 3.255 | 165 | 91.46 |
| 451 | Sahibzada Ajit Singh Nagar | 556 | 75.50 | 1.53 | 1.81 | 2.09 | 2.340 | 163 | 29.27 | 3.480 | 513 | 92.29 |
| 452 | Sahid Bhagat Singh Nagar | 174 | 64.70 | 1.34 | 1.58 | 1.82 | 1.969 | 43 | 24.61 | 2.767 | 131 | 75.12 |
| 453 | Sangrur | 380 | 65.60 | 1.32 | 1.59 | 1.86 | 1.987 | 95 | 24.99 | 2.806 | 291 | 76.49 |
| 454 | Tarn Taran | 159 | 80.80 | 1.45 | 1.70 | 1.96 | 2.237 | 50 | 31.62 | 3.422 | 161 | 101.32 |
|  | **Rajasthan** | |  |  |  |  |  |  |  |  |  |  |
| 455 | Ajmer | 502 | 68.70 | 1.95 | 2.14 | 2.33 | 2.703 | 132 | 26.31 | 3.880 | 408 | 81.29 |
| 456 | Alwar | 283 | 59.80 | 2.22 | 2.52 | 2.83 | 3.586 | 120 | 42.31 | 4.230 | 192 | 67.84 |
| 457 | Banswara | 286 | 54.90 | 2.21 | 2.51 | 2.81 | 3.470 | 109 | 38.25 | 4.038 | 174 | 60.87 |
| 458 | Baran | 236 | 65.40 | 1.72 | 1.97 | 2.22 | 2.461 | 59 | 24.90 | 3.471 | 179 | 76.18 |
| 459 | Barmer | 348 | 46.20 | 3.22 | 3.60 | 4.00 | 4.929 | 129 | 36.91 | 5.371 | 171 | 49.20 |
| 460 | Bharatpur | 379 | 44.60 | 3.17 | 3.53 | 3.91 | 4.781 | 134 | 35.43 | 5.194 | 179 | 47.14 |
| 461 | Bhilwara | 224 | 57.00 | 1.93 | 2.22 | 2.51 | 3.107 | 90 | 39.98 | 3.637 | 143 | 63.82 |
| 462 | Bikaner | 615 | 71.40 | 2.27 | 2.47 | 2.67 | 3.764 | 322 | 52.39 | 4.584 | 526 | 85.58 |
| 463 | Bundi | 237 | 57.70 | 2.19 | 2.52 | 2.85 | 3.542 | 96 | 40.56 | 4.153 | 153 | 64.82 |
| 464 | Chittaurgarh | 167 | 47.30 | 1.62 | 1.90 | 2.20 | 1.956 | 5 | 2.93 | 2.862 | 85 | 50.62 |
| 465 | Churu | 281 | 52.40 | 2.04 | 2.31 | 2.59 | 3.147 | 102 | 36.23 | 3.637 | 161 | 57.43 |
| 466 | Dausa | 257 | 54.80 | 2.03 | 2.31 | 2.61 | 3.192 | 98 | 38.17 | 3.713 | 156 | 60.73 |
| 467 | Dhaulpur | 353 | 53.70 | 2.79 | 3.12 | 3.46 | 4.283 | 131 | 37.28 | 4.967 | 209 | 59.21 |
| 468 | Dungarpur | 305 | 64.20 | 2.57 | 2.90 | 3.23 | 4.235 | 140 | 46.05 | 5.057 | 227 | 74.36 |
| 469 | Ganganagar | 239 | 71.10 | 1.63 | 1.87 | 2.12 | 2.381 | 65 | 27.35 | 3.461 | 203 | 85.10 |
| 470 | Hanumangarh | 182 | 70.60 | 1.59 | 1.83 | 2.09 | 2.326 | 49 | 27.13 | 3.373 | 154 | 84.30 |
| 471 | Jaipur | 479 | 66.70 | 1.85 | 2.03 | 2.21 | 2.547 | 122 | 25.46 | 3.617 | 374 | 78.18 |
| 472 | Jaisalmer | 506 | 53.50 | 2.87 | 3.22 | 3.58 | 4.415 | 188 | 37.12 | 5.118 | 298 | 58.93 |
| 473 | Jalor | 222 | 59.00 | 2.75 | 3.08 | 3.42 | 4.362 | 93 | 41.64 | 5.134 | 148 | 66.69 |
| 474 | Jhalawar | 195 | 68.20 | 1.60 | 1.86 | 2.13 | 2.345 | 51 | 26.10 | 3.357 | 157 | 80.51 |
| 475 | Jhunjhunun | 324 | 63.70 | 1.60 | 1.84 | 2.08 | 2.285 | 78 | 24.18 | 3.194 | 238 | 73.61 |
| 476 | Jodhpur | 236 | 61.20 | 2.15 | 2.35 | 2.55 | 3.372 | 102 | 43.49 | 3.992 | 165 | 69.89 |
| 477 | Karauli | 167 | 56.00 | 2.65 | 2.99 | 3.33 | 4.161 | 65 | 39.15 | 4.856 | 104 | 62.41 |
| 478 | Kota | 98 | 71.30 | 1.55 | 1.72 | 1.90 | 2.192 | 27 | 27.43 | 3.189 | 83 | 85.42 |
| 479 | Nagaur | 170 | 54.70 | 1.87 | 2.14 | 2.41 | 2.577 | 35 | 20.44 | 3.437 | 103 | 60.59 |
| 480 | Pali | 280 | 57.10 | 1.91 | 2.20 | 2.50 | 2.671 | 60 | 21.43 | 3.607 | 179 | 63.97 |
| 481 | Pratapgarh | 248 | 63.60 | 2.27 | 2.58 | 2.89 | 3.755 | 113 | 45.53 | 4.475 | 182 | 73.46 |
| 482 | Rajsamand | 114 | 61.20 | 2.42 | 2.76 | 3.10 | 3.960 | 50 | 43.49 | 4.689 | 80 | 69.89 |
| 483 | Sawai Madhopur | 193 | 50.40 | 2.38 | 2.69 | 3.00 | 3.622 | 67 | 34.63 | 4.162 | 106 | 54.72 |
| 484 | Sikar | 81 | 59.80 | 1.96 | 2.22 | 2.48 | 3.159 | 34 | 42.31 | 3.726 | 55 | 67.84 |
| 485 | Sirohi | 398 | 47.50 | 2.66 | 3.00 | 3.34 | 4.144 | 152 | 38.13 | 4.527 | 203 | 50.89 |
| 486 | Tonk | 234 | 66.00 | 1.91 | 2.20 | 2.48 | 2.753 | 59 | 25.16 | 3.896 | 181 | 77.10 |
| 487 | Udaipur | 217 | 51.20 | 2.49 | 2.84 | 3.19 | 3.842 | 77 | 35.27 | 4.425 | 121 | 55.80 |
|  | **Sikkim** |  |  |  |  |  |  |  |  |  |  |  |
| 488 | East District | 267 | 32.10 | 1.10 | 1.25 | 1.41 | 1.275 | 5 | 1.98 | 1.651 | 85 | 32.05 |
| 489 | North District | 124 | 50.20 | 1.09 | 1.31 | 1.53 | 1.554 | 23 | 18.61 | 2.023 | 68 | 54.45 |
| 490 | Sauth District | 330 | 59.30 | 0.89 | 1.08 | 1.28 | 1.321 | 74 | 22.34 | 1.805 | 222 | 67.12 |
| 491 | West District | 212 | 65.80 | 0.86 | 1.05 | 1.25 | 1.313 | 53 | 25.07 | 1.856 | 163 | 76.80 |
|  | **Tamil Nadu** | |  |  |  |  |  |  |  |  |  |  |
| 492 | Ariyalur | 130 | 35.90 | 1.41 | 1.70 | 2.00 | 1.738 | 3 | 2.21 | 2.320 | 47 | 36.46 |
| 493 | Chennai | 99 | 60.10 | 0.79 | 0.99 | 1.19 | 1.214 | 22 | 22.67 | 1.666 | 67 | 68.28 |
| 494 | Coimbatore | 155 | 65.40 | 1.50 | 1.78 | 2.06 | 2.223 | 39 | 24.90 | 3.136 | 118 | 76.18 |
| 495 | Cuddalore | 178 | 55.50 | 1.60 | 1.88 | 2.16 | 2.270 | 37 | 20.77 | 3.040 | 110 | 61.71 |
| 496 | Dharmapuri | 164 | 55.00 | 1.47 | 1.73 | 2.00 | 2.086 | 34 | 20.56 | 2.785 | 100 | 61.01 |
| 497 | Dindigul | 164 | 60.30 | 1.47 | 1.73 | 2.00 | 2.124 | 37 | 22.75 | 2.916 | 112 | 68.57 |
| 498 | Erode | 102 | 63.00 | 1.40 | 1.66 | 1.93 | 2.057 | 24 | 23.89 | 2.865 | 74 | 72.56 |
| 499 | Kancheepuram | 134 | 61.60 | 1.16 | 1.39 | 1.64 | 1.714 | 31 | 23.30 | 2.370 | 95 | 70.48 |
| 500 | Kanniakumari | 314 | 45.10 | 1.66 | 1.96 | 2.27 | 2.015 | 9 | 2.79 | 2.897 | 150 | 47.78 |
| 501 | Karur | 224 | 57.60 | 1.69 | 1.98 | 2.28 | 2.408 | 49 | 21.63 | 3.261 | 145 | 64.68 |
| 502 | Krishnagiri | 217 | 60.80 | 1.49 | 1.74 | 2.00 | 2.140 | 50 | 22.96 | 2.946 | 150 | 69.30 |
| 503 | Madurai | 322 | 44.50 | 1.50 | 1.82 | 2.14 | 1.870 | 9 | 2.75 | 2.676 | 151 | 47.02 |
| 504 | Nagapattinam | 248 | 57.40 | 1.31 | 1.57 | 1.84 | 1.908 | 53 | 21.55 | 2.581 | 160 | 64.39 |
| 505 | Namakal | 244 | 59.40 | 1.29 | 1.55 | 1.83 | 1.897 | 55 | 22.38 | 2.593 | 164 | 67.26 |
| 506 | Perambalur | 250 | 41.00 | 1.33 | 1.60 | 1.87 | 1.641 | 6 | 2.53 | 2.282 | 106 | 42.63 |
| 507 | Pudukottai | 165 | 40.60 | 1.67 | 1.96 | 2.26 | 2.009 | 4 | 2.51 | 2.786 | 69 | 42.13 |
| 508 | Ramanathapuram | 274 | 26.00 | 1.76 | 2.09 | 2.42 | 2.123 | 4 | 1.60 | 2.618 | 69 | 25.25 |
| 509 | Salem | 204 | 52.10 | 1.55 | 1.82 | 2.10 | 2.173 | 40 | 19.38 | 2.858 | 117 | 57.02 |
| 510 | Sivaganga | 372 | 43.30 | 1.64 | 1.94 | 2.24 | 1.992 | 10 | 2.68 | 2.823 | 169 | 45.50 |
| 511 | Thanjavur | 262 | 48.50 | 1.62 | 1.92 | 2.23 | 1.978 | 8 | 3.00 | 2.922 | 137 | 52.20 |
| 512 | The Nilgiris | 107 | 56.50 | 1.31 | 1.56 | 1.83 | 1.890 | 23 | 21.18 | 2.545 | 68 | 63.12 |
| 513 | Theni | 124 | 38.50 | 1.24 | 1.53 | 1.82 | 1.566 | 3 | 2.38 | 2.135 | 49 | 39.57 |
| 514 | Thiruvallur | 105 | 64.00 | 1.19 | 1.45 | 1.71 | 1.802 | 26 | 24.31 | 2.524 | 78 | 74.06 |
| 515 | Thiruvarur | 124 | 55.30 | 1.58 | 1.87 | 2.17 | 2.257 | 26 | 20.69 | 3.019 | 76 | 61.43 |
| 516 | Tiruchirapalli | 144 | 43.40 | 1.56 | 1.86 | 2.17 | 1.910 | 4 | 2.68 | 2.709 | 66 | 45.62 |
| 517 | Tirunelveli | 107 | 36.10 | 1.35 | 1.67 | 1.99 | 1.707 | 2 | 2.23 | 2.283 | 39 | 36.70 |
| 518 | Tiruppur | 149 | 63.10 | 1.38 | 1.64 | 1.92 | 2.032 | 36 | 23.93 | 2.832 | 108 | 72.71 |
| 519 | Tiruvannamalai | 160 | 48.90 | 1.46 | 1.73 | 2.00 | 1.782 | 5 | 3.03 | 2.642 | 85 | 52.73 |
| 520 | Toothukkudi | 258 | 30.10 | 1.43 | 1.74 | 2.05 | 1.772 | 5 | 1.85 | 2.258 | 77 | 29.78 |
| 521 | Vellore | 269 | 64.00 | 1.54 | 1.80 | 2.08 | 2.238 | 65 | 24.31 | 3.133 | 199 | 74.06 |
| 522 | Viluppuram | 128 | 49.90 | 1.55 | 1.81 | 2.08 | 1.866 | 4 | 3.09 | 2.788 | 69 | 54.05 |
| 523 | Virudhunagar | 112 | 23.30 | 1.63 | 1.97 | 2.32 | 1.998 | 2 | 1.43 | 2.410 | 25 | 22.36 |
|  | **Telangana** | |  |  |  |  |  |  |  |  |  |  |
| 524 | Adilabad | 151 | 48.70 | 1.55 | 1.84 | 2.14 | 1.895 | 5 | 3.02 | 2.805 | 79 | 52.46 |
| 525 | Hyderabad | 125 | 56.50 | 1.55 | 1.82 | 2.10 | 2.205 | 26 | 21.18 | 2.969 | 79 | 63.12 |
| 526 | Karimnagar | 314 | 38.60 | 1.15 | 1.42 | 1.70 | 1.454 | 7 | 2.38 | 1.984 | 125 | 39.69 |
| 527 | Khammam | 463 | 69.10 | 1.38 | 1.66 | 1.94 | 2.100 | 123 | 26.48 | 3.020 | 380 | 81.92 |
| 528 | Mahbubnagar | 145 | 64.30 | 2.17 | 2.52 | 2.88 | 3.683 | 67 | 46.14 | 4.398 | 108 | 74.51 |
| 529 | Medak | 209 | 49.60 | 1.45 | 1.73 | 2.03 | 1.783 | 6 | 3.07 | 2.658 | 112 | 53.65 |
| 530 | Nalgonda | 131 | 68.00 | 1.52 | 1.82 | 2.13 | 2.293 | 34 | 26.01 | 3.280 | 105 | 80.20 |
| 531 | Nizamabad | 134 | 47.00 | 1.73 | 2.05 | 2.38 | 2.110 | 4 | 2.91 | 3.080 | 67 | 50.23 |
| 532 | Rangareddy | 245 | 69.10 | 1.37 | 1.65 | 1.93 | 2.087 | 65 | 26.48 | 3.002 | 200 | 81.92 |
| 533 | Warangal | 118 | 50.80 | 1.12 | 1.39 | 1.66 | 1.652 | 22 | 18.85 | 2.158 | 65 | 55.26 |
|  | **Tripura** |  |  |  |  |  |  |  |  |  |  |  |
| 534 | Dhalai | 163 | 67.10 | 1.43 | 1.69 | 1.96 | 2.123 | 42 | 25.63 | 3.022 | 128 | 78.80 |
| 535 | North Tripura | 210 | 65.20 | 2.03 | 2.35 | 2.67 | 3.452 | 98 | 46.91 | 4.133 | 159 | 75.88 |
| 536 | South Tripura | 126 | 66.90 | 1.13 | 1.37 | 1.62 | 1.720 | 32 | 25.54 | 2.445 | 99 | 78.49 |
| 537 | West Tripura | 298 | 61.90 | 1.39 | 1.56 | 1.75 | 1.925 | 70 | 23.42 | 2.666 | 211 | 70.92 |
|  | **Uttar Pradesh** | |  |  |  |  |  |  |  |  |  |  |
| 538 | Agra | 668 | 60.80 | 2.58 | 2.80 | 3.01 | 4.008 | 288 | 43.15 | 4.741 | 463 | 69.30 |
| 539 | Aligarh | 692 | 58.70 | 2.63 | 2.85 | 3.07 | 4.030 | 287 | 41.39 | 4.738 | 459 | 66.25 |
| 540 | Allahabad | 281 | 37.70 | 2.16 | 2.46 | 2.75 | 3.179 | 82 | 29.22 | 3.410 | 108 | 38.61 |
| 541 | Ambedkar Nagar | 304 | 35.60 | 2.09 | 2.36 | 2.63 | 3.006 | 83 | 27.39 | 3.212 | 110 | 36.11 |
| 542 | Auraiya | 246 | 44.50 | 2.26 | 2.60 | 2.93 | 3.519 | 87 | 35.34 | 3.822 | 116 | 47.02 |
| 543 | Azamgarh | 278 | 37.10 | 2.16 | 2.45 | 2.75 | 3.153 | 80 | 28.70 | 3.378 | 105 | 37.89 |
| 544 | Baghpat | 262 | 68.20 | 1.96 | 2.24 | 2.51 | 3.350 | 130 | 49.54 | 4.043 | 211 | 80.51 |
| 545 | Bahraich | 404 | 10.70 | 3.79 | 4.22 | 4.65 | 4.538 | 30 | 7.55 | 4.630 | 39 | 9.71 |
| 546 | Ballia | 327 | 32.90 | 2.53 | 2.84 | 3.16 | 3.552 | 82 | 25.07 | 3.776 | 108 | 32.96 |
| 547 | Balrampur | 394 | 2.70 | 3.03 | 3.38 | 3.72 | 3.443 | 7 | 1.85 | 3.460 | 9 | 2.37 |
| 548 | Banda | 188 | 54.40 | 2.29 | 2.67 | 3.06 | 3.680 | 71 | 37.85 | 4.277 | 113 | 60.18 |
| 549 | Barabanki | 241 | 37.90 | 2.27 | 2.60 | 2.94 | 3.364 | 71 | 29.40 | 3.610 | 94 | 38.85 |
| 550 | Bareilly | 556 | 64.90 | 2.31 | 2.52 | 2.74 | 3.696 | 259 | 46.65 | 4.421 | 419 | 75.42 |
| 551 | Basti | 334 | 18.30 | 2.68 | 3.01 | 3.34 | 3.409 | 44 | 13.25 | 3.527 | 57 | 17.17 |
| 552 | Bijnor | 320 | 53.00 | 2.43 | 2.74 | 3.05 | 3.746 | 118 | 36.71 | 4.336 | 187 | 58.25 |
| 553 | Budaun | 386 | 51.60 | 3.35 | 3.73 | 4.11 | 5.057 | 138 | 35.59 | 5.831 | 218 | 56.34 |
| 554 | Bulandshahar | 351 | 57.80 | 2.61 | 2.92 | 3.23 | 4.107 | 143 | 40.64 | 4.817 | 228 | 64.96 |
| 555 | Chandauli | 355 | 38.30 | 2.46 | 2.75 | 3.04 | 3.568 | 106 | 29.75 | 3.832 | 140 | 39.33 |
| 556 | Chitrakoot | 331 | 45.80 | 2.99 | 3.36 | 3.73 | 4.588 | 121 | 36.54 | 4.996 | 161 | 48.68 |
| 557 | Deoria | 302 | 32.30 | 2.15 | 2.43 | 2.71 | 3.027 | 74 | 24.56 | 3.214 | 98 | 32.28 |
| 558 | Etah | 157 | 54.50 | 2.69 | 3.02 | 3.35 | 4.165 | 60 | 37.93 | 4.842 | 95 | 60.31 |
| 559 | Etawah | 334 | 51.40 | 2.21 | 2.51 | 2.82 | 3.399 | 118 | 35.43 | 3.917 | 187 | 56.07 |
| 560 | Faizabad | 283 | 43.80 | 2.31 | 2.63 | 2.94 | 3.542 | 98 | 34.69 | 3.843 | 130 | 46.13 |
| 561 | Farrukhabad | 139 | 45.80 | 2.89 | 3.24 | 3.58 | 4.424 | 51 | 36.54 | 4.817 | 68 | 48.68 |
| 562 | Fatehpur | 129 | 41.40 | 1.98 | 2.32 | 2.67 | 3.074 | 42 | 32.51 | 3.320 | 56 | 43.12 |
| 563 | Firozabad | 187 | 47.30 | 2.56 | 2.78 | 3.00 | 3.835 | 71 | 37.94 | 4.187 | 95 | 50.62 |
| 564 | Gautam Buddha Nagar | 161 | 74.60 | 2.40 | 2.61 | 2.81 | 4.053 | 89 | 55.29 | 4.980 | 147 | 90.80 |
| 565 | Ghaziabad | 471 | 65.90 | 2.21 | 2.42 | 2.62 | 3.570 | 224 | 47.52 | 4.282 | 363 | 76.95 |
| 566 | Ghazipur | 550 | 35.60 | 2.48 | 2.80 | 3.13 | 3.567 | 151 | 27.39 | 3.811 | 199 | 36.11 |
| 567 | Gonda | 182 | 13.80 | 2.97 | 3.31 | 3.65 | 3.636 | 18 | 9.84 | 3.730 | 23 | 12.69 |
| 568 | Gorakhpur | 332 | 45.00 | 2.10 | 2.38 | 2.66 | 3.232 | 119 | 35.80 | 3.514 | 158 | 47.65 |
| 569 | Hamirpur | 134 | 43.00 | 1.99 | 2.34 | 2.69 | 3.135 | 46 | 33.96 | 3.396 | 60 | 45.12 |
| 570 | Hardoi | 267 | 26.40 | 2.66 | 3.03 | 3.39 | 3.626 | 52 | 19.66 | 3.808 | 68 | 25.69 |
| 571 | Jalaun | 137 | 47.40 | 1.70 | 2.00 | 2.30 | 2.059 | 4 | 2.93 | 3.015 | 69 | 50.75 |
| 572 | Jaunpur | 247 | 38.10 | 2.42 | 2.72 | 3.02 | 3.524 | 73 | 29.57 | 3.783 | 96 | 39.09 |
| 573 | Jhansi | 192 | 65.90 | 1.84 | 2.05 | 2.25 | 2.565 | 48 | 25.11 | 3.627 | 148 | 76.95 |
| 574 | Jyotiba Phule Nagar | 160 | 59.40 | 2.62 | 2.95 | 3.27 | 4.188 | 67 | 41.97 | 4.934 | 107 | 67.26 |
| 575 | Kannauj | 123 | 36.50 | 2.72 | 3.06 | 3.41 | 3.922 | 35 | 28.17 | 4.198 | 46 | 37.18 |
| 576 | Kanpur Dehat | 109 | 47.70 | 2.22 | 2.54 | 2.86 | 3.513 | 42 | 38.31 | 3.839 | 56 | 51.15 |
| 577 | Kanpur Nagar | 244 | 59.80 | 1.45 | 1.64 | 1.82 | 2.010 | 55 | 22.55 | 2.753 | 165 | 67.84 |
| 578 | Kanshiram Nagar | 307 | 60.00 | 3.13 | 3.50 | 3.86 | 4.987 | 130 | 42.48 | 5.885 | 209 | 68.14 |
| 579 | Kaushambi | 212 | 35.30 | 2.88 | 3.27 | 3.65 | 4.157 | 57 | 27.13 | 4.439 | 76 | 35.76 |
| 580 | Kheri | 312 | 30.80 | 2.99 | 3.38 | 3.76 | 4.167 | 73 | 23.30 | 4.413 | 95 | 30.57 |
| 581 | Kushinagar | 92 | 29.30 | 2.69 | 3.00 | 3.32 | 3.661 | 20 | 22.05 | 3.867 | 27 | 28.88 |
| 582 | Lalitpur | 268 | 68.80 | 2.00 | 2.31 | 2.62 | 3.467 | 134 | 50.07 | 4.191 | 218 | 81.45 |
| 583 | Lucknow | 154 | 51.60 | 1.39 | 1.58 | 1.76 | 1.883 | 30 | 19.18 | 2.470 | 87 | 56.34 |
| 584 | Mahamaya Nagar | 344 | 60.10 | 2.43 | 2.74 | 3.05 | 3.906 | 146 | 42.56 | 4.611 | 235 | 68.28 |
| 585 | Maharajganj | 231 | 27.60 | 2.51 | 2.82 | 3.12 | 3.402 | 48 | 20.64 | 3.581 | 62 | 27.00 |
| 586 | Mahoba | 221 | 64.00 | 2.10 | 2.43 | 2.76 | 3.545 | 101 | 45.88 | 4.230 | 163 | 74.06 |
| 587 | Mainpuri | 276 | 40.50 | 2.37 | 2.69 | 3.02 | 3.543 | 88 | 31.71 | 3.820 | 116 | 42.01 |
| 588 | Mathura | 310 | 57.10 | 2.56 | 2.88 | 3.20 | 4.034 | 124 | 40.06 | 4.722 | 198 | 63.97 |
| 589 | Mau | 332 | 34.80 | 2.36 | 2.66 | 2.96 | 3.370 | 89 | 26.70 | 3.596 | 117 | 35.17 |
| 590 | Meerut | 142 | 65.30 | 2.10 | 2.27 | 2.43 | 3.337 | 67 | 47.00 | 3.996 | 108 | 76.03 |
| 591 | Mirzapur | 318 | 48.60 | 2.59 | 2.91 | 3.23 | 4.050 | 124 | 39.16 | 4.433 | 166 | 52.33 |
| 592 | Moradabad | 648 | 61.00 | 2.72 | 2.95 | 3.18 | 4.228 | 281 | 43.32 | 5.003 | 451 | 69.60 |
| 593 | Muzaffarnagar | 330 | 62.50 | 2.77 | 3.10 | 3.43 | 4.482 | 147 | 44.59 | 5.326 | 237 | 71.82 |
| 594 | Pilibhit | 169 | 62.90 | 2.41 | 2.73 | 3.04 | 3.957 | 76 | 44.93 | 4.707 | 122 | 72.41 |
| 595 | Pratapgarh | 161 | 38.30 | 2.01 | 2.30 | 2.59 | 2.984 | 48 | 29.75 | 3.205 | 63 | 39.33 |
| 596 | Rai Bareili | 224 | 27.80 | 2.15 | 2.48 | 2.80 | 2.996 | 47 | 20.81 | 3.155 | 61 | 27.22 |
| 597 | Rampur | 342 | 62.50 | 2.61 | 2.94 | 3.25 | 4.251 | 152 | 44.59 | 5.051 | 245 | 71.82 |
| 598 | Saharanpur | 468 | 65.00 | 2.51 | 2.72 | 2.94 | 3.991 | 219 | 46.74 | 4.776 | 354 | 75.58 |
| 599 | Sant Kabirnagar | 246 | 21.90 | 2.72 | 3.05 | 3.37 | 3.540 | 39 | 16.06 | 3.687 | 51 | 20.88 |
| 600 | Sant Ravidasnagar | 355 | 43.00 | 2.68 | 3.00 | 3.31 | 4.019 | 121 | 33.96 | 4.354 | 160 | 45.12 |
| 601 | Shahjahanpur | 434 | 50.60 | 3.10 | 3.48 | 3.86 | 4.691 | 151 | 34.79 | 5.394 | 239 | 54.99 |
| 602 | Shrawasti | 384 | 8.40 | 3.96 | 4.40 | 4.84 | 4.659 | 23 | 5.88 | 4.732 | 29 | 7.55 |
| 603 | Siddhartha Nagar | 284 | 27.80 | 3.05 | 3.41 | 3.76 | 4.120 | 59 | 20.81 | 4.338 | 77 | 27.22 |
| 604 | Sitapur | 192 | 42.80 | 2.94 | 3.32 | 3.71 | 4.442 | 65 | 33.78 | 4.810 | 86 | 44.87 |
| 605 | Sonabhadra | 179 | 44.50 | 2.50 | 2.83 | 3.17 | 3.830 | 63 | 35.34 | 4.161 | 84 | 47.02 |
| 606 | Sultanpur | 374 | 32.30 | 2.41 | 2.74 | 3.07 | 3.413 | 92 | 24.56 | 3.624 | 121 | 32.28 |
| 607 | Unnao | 82 | 41.80 | 2.41 | 2.74 | 3.07 | 3.641 | 27 | 32.88 | 3.935 | 36 | 43.62 |
| 608 | Varanasi | 176 | 58.50 | 2.03 | 2.22 | 2.41 | 3.135 | 73 | 41.22 | 3.684 | 116 | 65.97 |
|  | **Uttarakhand** | |  |  |  |  |  |  |  |  |  |  |
| 609 | Almora | 221 | 57.10 | 2.07 | 2.39 | 2.71 | 3.347 | 88 | 40.06 | 3.919 | 141 | 63.97 |
| 610 | Bageshwar | 203 | 57.90 | 1.75 | 2.03 | 2.31 | 2.472 | 44 | 21.76 | 3.352 | 132 | 65.11 |
| 611 | Chamoli | 191 | 60.20 | 1.65 | 1.94 | 2.23 | 2.381 | 43 | 22.71 | 3.267 | 131 | 68.43 |
| 612 | Champawat | 549 | 63.30 | 1.77 | 2.07 | 2.36 | 2.567 | 132 | 24.01 | 3.581 | 401 | 73.01 |
| 613 | Dehradun | 310 | 59.80 | 1.32 | 1.49 | 1.65 | 1.826 | 70 | 22.55 | 2.501 | 210 | 67.84 |
| 614 | Garhwal | 356 | 67.20 | 1.67 | 1.98 | 2.28 | 2.488 | 91 | 25.67 | 3.543 | 281 | 78.95 |
| 615 | Hardwar | 277 | 41.50 | 2.55 | 2.78 | 3.01 | 3.686 | 90 | 32.60 | 3.982 | 120 | 43.25 |
| 616 | Nainital | 317 | 48.50 | 1.77 | 1.96 | 2.15 | 2.019 | 10 | 3.00 | 2.983 | 165 | 52.20 |
| 617 | Pithoragarh | 161 | 70.10 | 1.75 | 2.06 | 2.37 | 2.614 | 43 | 26.91 | 3.780 | 134 | 83.50 |
| 618 | Rudra Prayag | 148 | 61.00 | 1.70 | 2.00 | 2.30 | 2.461 | 34 | 23.05 | 3.392 | 103 | 69.60 |
| 619 | Tehri Garhwal | 140 | 64.30 | 1.68 | 1.95 | 2.22 | 2.426 | 34 | 24.44 | 3.403 | 104 | 74.51 |
| 620 | Udham Singh Nagar | 253 | 38.00 | 1.97 | 2.17 | 2.36 | 2.221 | 6 | 2.35 | 3.016 | 99 | 38.97 |
| 621 | Uttarkashi | 162 | 68.50 | 1.81 | 2.09 | 2.38 | 2.638 | 43 | 26.23 | 3.782 | 131 | 80.98 |
|  | **West Bengal** | |  |  |  |  |  |  |  |  |  |  |
| 622 | Bankura | 155 | 76.30 | 1.45 | 1.72 | 1.99 | 2.229 | 46 | 29.62 | 3.330 | 145 | 93.63 |
| 623 | Barddhaman | 154 | 77.10 | 1.30 | 1.55 | 1.80 | 2.015 | 46 | 29.97 | 3.022 | 146 | 94.97 |
| 624 | Birbhum | 165 | 77.10 | 1.47 | 1.74 | 2.01 | 2.261 | 49 | 29.97 | 3.393 | 156 | 94.97 |
| 625 | Dakshin Dinajpur | 147 | 60.30 | 1.40 | 1.67 | 1.94 | 2.050 | 33 | 22.75 | 2.815 | 101 | 68.57 |
| 626 | Darjiling | 133 | 73.40 | 1.27 | 1.53 | 1.79 | 1.964 | 38 | 28.35 | 2.889 | 118 | 88.82 |
| 627 | Haora | 211 | 67.50 | 1.34 | 1.60 | 1.85 | 2.013 | 55 | 25.80 | 2.871 | 168 | 79.42 |
| 628 | Hugli | 149 | 77.90 | 1.25 | 1.52 | 1.78 | 1.981 | 45 | 30.32 | 2.984 | 144 | 96.33 |
| 629 | Jalpaiguri | 339 | 49.10 | 1.42 | 1.69 | 1.97 | 1.741 | 10 | 3.04 | 2.586 | 179 | 52.99 |
| 630 | Koch Bihar | 147 | 65.70 | 1.56 | 1.85 | 2.13 | 2.313 | 37 | 25.03 | 3.268 | 113 | 76.64 |
| 631 | Kolkata | 97 | 70.00 | 1.14 | 1.41 | 1.68 | 1.789 | 26 | 26.87 | 2.585 | 81 | 83.35 |
| 632 | Maldah | 151 | 60.30 | 2.12 | 2.44 | 2.76 | 3.483 | 65 | 42.73 | 4.113 | 104 | 68.57 |
| 633 | Murshidabad | 359 | 72.80 | 1.69 | 1.96 | 2.23 | 2.510 | 101 | 28.08 | 3.682 | 316 | 87.85 |
| 634 | Nadia | 272 | 74.30 | 1.53 | 1.81 | 2.09 | 2.330 | 78 | 28.74 | 3.444 | 245 | 90.30 |
| 635 | North 24 Paragnas | 98 | 73.00 | 1.20 | 1.46 | 1.72 | 1.871 | 28 | 28.17 | 2.747 | 87 | 88.17 |
| 636 | Pashchim Medinipur | 178 | 73.80 | 1.34 | 1.60 | 1.85 | 2.056 | 51 | 28.52 | 3.032 | 160 | 89.48 |
| 637 | Purba Medinipur | 328 | 75.30 | 1.45 | 1.72 | 1.99 | 2.222 | 96 | 29.18 | 3.302 | 302 | 91.96 |
| 638 | Puruliya | 239 | 69.00 | 1.97 | 2.26 | 2.55 | 3.396 | 120 | 50.25 | 4.108 | 196 | 81.76 |
| 639 | South 24 Paragnas | 155 | 68.60 | 1.80 | 2.09 | 2.38 | 2.639 | 41 | 26.27 | 3.786 | 126 | 81.14 |
| 640 | Uttar Dinajpur | 130 | 54.70 | 2.32 | 2.65 | 2.97 | 3.659 | 50 | 38.09 | 4.256 | 79 | 60.59 |
